# Trisomy 21-driven metabolite alterations are linked to cellular injuries in Down syndrome

**DOI:** 10.1007/s00018-024-05127-0

**Published:** 2024-03-03

**Authors:** Juli Liu, Shaoxian Chen, Guiping Huang, Pengju Wen, Xianwu Zhou, Yueheng Wu

**Affiliations:** 1 Medical Research Institute, Guangdong Provincial People’s Hospital (Guangdong Academy of Medical Sciences), Southern Medical University, Guangzhou, 510080 Guangdong China; 2 Guangdong Provincial Key Laboratory of South China Structural Heart Disease, Guangdong Cardiovascular Institute, Guangdong Provincial People’s Hospital (Guangdong Academy of Medical Sciences), Southern Medical University, Guangzhou, 510080 Guangdong China; 3 Department of Cardiovascular Surgery, Guangdong Cardiovascular Institute, Guangdong Provincial People’s Hospital (Guangdong Academy of Medical Sciences), Southern Medical University, Guangzhou, 510080 Guangdong China; 4 Department of Thoracic and Cardiovascular Surgery, Zhongnan Hospital of Wuhan University, Wuhan, 430071 Hubei China

**Keywords:** DNA damage, Cardiac development, 5-oxo-ETE, Calcitriol, Congenital heart disease

## Abstract

Down syndrome (DS) arises from a genetic anomaly characterized by an extra copy of chromosome 21 (exCh21). Despite high incidence of congenital diseases among DS patients, direct impacts of exCh21 remain elusive. Here, we established a robust DS model harnessing human-induced pluripotent stem cells (hiPSCs) from mosaic DS patient. These hiPSC lines encompassed both those with standard karyotype and those carrying an extra copy of exCh21, allowing to generate isogenic cell lines with a consistent genetic background. We unraveled that exCh21 inflicted disruption upon the cellular transcriptome, ushering in alterations in metabolic processes and triggering DNA damage. The impact of exCh21 was also manifested in profound modifications in chromatin accessibility patterns. Moreover, we identified two signature metabolites, 5-oxo-ETE and Calcitriol, whose biosynthesis is affected by exCh21. Notably, supplementation with 5-oxo-ETE promoted DNA damage, in stark contrast to the protective effect elicited by Calcitriol against such damage. We also found that exCh21 disrupted cardiogenesis, and that this impairment could be mitigated through supplementation with Calcitriol. Specifically, the deleterious effects of 5-oxo-ETE unfolded in the form of DNA damage induction and the repression of cardiogenesis. On the other hand, Calcitriol emerged as a potent activator of its nuclear receptor VDR, fostering amplified binding to chromatin and subsequent facilitation of gene transcription. Our findings provide a comprehensive understanding of exCh21’s metabolic implications within the context of Down syndrome, offering potential avenues for therapeutic interventions for Down syndrome treatment.

**Supplementary Information:**

The online version contains supplementary material available at 10.1007/s00018-024-05127-0.

## Introduction

Down syndrome or Down’s syndrome (DS), which is also called trisomy 21, is a genetic disorder caused by the presence of extra copy of chromosome 21 (exCh21). Trisomy 21 is the most common viable human aneuploidy [1]. Individuals with DS exhibit various abnormal health conditions, including congenital heart disease (CHD) and neurological complications [2–4].

Although the fundamental chromosomal basis of DS has been well understood for an extended period [5], the precise mechanisms through which the surplus exCh21 precipitates congenital disorders in humans have remained enigmatic [6]. This ambiguity might partly stem from the challenges in sourcing appropriate patient materials and the limitations inherent in animal models that struggle to fully replicate the complex phenotypes seen in human trisomy 21 [7, 8]. As a result, human-induced pluripotent stem cells (hiPSCs) that are derived from DS patients offer a promising avenue for creating an *in vitro* model of DS, thereby enabling the exploration of its underlying molecular mechanisms [7, 9]. Indeed, accumulating evidence suggests that hiPSC models are instrumental in delineating the pathogenesis of DS [9]. Furthermore, the differentiation of hiPSCs obtained from DS patients proves especially valuable for scrutinizing congenital maladies in human development. However, the precise mechanisms governing hiPSCs and their developmental processes remain inadequately understood.

The direct influence of exCh21 on the human cardiac system remains elusive, despite much higher incidence of congenital heart diseases (CHDs) among DS patients [2, 10, 11]. CHD occurs in approximately 50% of newborns identified with DS, making it a prevalent condition among individuals with DS [12]. Consequently, cardiac issues are highly frequent in this population, encompassing a range of CHD like atrial septal defect (ASD) and ventricular septal defect (VSD) [12]. While there is a comprehensive description of cardiovascular conditions related to DS, there is a notable absence of in-depth, DS-specific expert perspectives concerning the recognition, diagnosis, and management of cardiovascular diseases [12]. This gap arises from the intricate mechanisms underlying DS that are not yet fully understood.

Our research objectives encompassed delving into the pathogenesis of DS through an *in vitro* approach using human-induced pluripotent stem cells (hiPSCs). The hiPSC lines were established from mosaic DS patient, yielding both cell lines with a regular karyotype and those harboring an extra of chromosome 21 (exCh21). This model allowed for the generation of isogenic cell lines with a consistent genetic background, effectively isolating the impact of the extra chromosome 21 (exCh21) on cellular processes. To unravel the intricate molecular mechanisms underpinning DS, we undertook multi-omics analyses to ascertain whether the presence of exCh21 directly triggers disorders within hiPSCs and hiPSC-derived cardiac lineages. These analyses unveiled abnormal metabolic patterns instigated by the additional copy of chromosome 21 in DS. Through our study, we not only identified potential biomarkers but also characterized the atypical metabolic traits associated with DS. Our findings offer significant insights into the origins of DS and present prospective avenues for treating both DS itself and the congenital heart disease (CHD) that stems from it.

## Materials and methods

### Human-induced pluripotent stem cells

The Institutional Review Board (IRB) at Guangdong Provincial People’s Hospital and Guangdong Academy of Medical Sciences (Guangzhou, China) approved the protocols utilized in this research. Consent in written form was acquired from the donors participating in the study. For the reprogramming of human-induced pluripotent stem cells (hiPSCs), fresh urine was collected from a mosaic Down’s syndrome patient. The urine was used to isolate live cells following a previously published protocol [13]. The obtained human urine-derived cells were then reprogrammed into iPSC lines. Individual clones were selected, and these clones were thoroughly validated using the protocol established by the South China Institute for Stem Cell Biology and Regenerative Medicine, Chinese Academy of Sciences (Guangzhou, China), which was detailed in the published protocol [13]. To ensure consistency and minimize genetic variability, three P hiPSC lines and three paired isogenic N hiPSC lines were chosen, expanded, and employed in the study. This selection aimed to avoid discrepancies stemming from differing genetic backgrounds.

### Human hESCs and iPSCs culture

The H9 cell line of human embryonic stem cells (hESCs) and the N and P hiPSC lines derived from mosaic Down’s syndrome patient was regularly nurtured in mTesR1 medium. These cells were cultured on 6-well plates coated with Matrigel. When the hiPSCs reached confluency, they were passaged using the ReLeSR Human PSC Selection and Passaging Reagent. The process involved initial washing of the confluently grown hiPSCs with 1 × phosphate-buffered saline (PBS). Subsequently, the cells were exposed to ReLeSR at room temperature (RT) for 1 minute and then placed in a 37 °C incubator for 7 min. After this, the cells were resuspended in mTesR1 medium and expanded into 6-well plates at a 1:10 ratio. To enhance cell viability during passaging, a ROCK inhibitor called Y27632 was added to the mTesR1 medium. The concentration of Y27632 in the mTesR1 medium was maintained at 10 μM. Following a 24-h incubation period, the medium containing Y27632 was replaced with fresh mTesR1 medium devoid of Y27632. This medium renewal process was repeated daily until the subsequent passaging of the cells.

### Cardiac differentiation

The process of cardiac differentiation was conducted using the STEMdiff Cardiomyocyte Differentiation Kit (STEM CELL Technologies), following the instructions outlined in the manual. Initially, hESCs or hiPSCs were dissociated and then re-seeded onto Matrigel-coated 12-well plates. Once the cells reached full confluence, they were exposed to differentiation basal medium supplemented with supplement A. After 48 h, the medium was substituted with differentiation basal medium containing supplement B. Moving forward, on day 4, the cells were incubated in differentiation basal medium along with supplement C. This medium was refreshed every 2 days. Upon reaching day 8, the cells were maintained in STEMdiff™ cardiomyocyte maintenance medium supplemented with supplement M. Similar to previous stages, this medium was renewed every 2 days. For experiments focused on human cardiac development, cells at specific time points in the differentiation process were collected as dictated by the experimental designs.

### Immunostaining

Cells were fixed using a 4% paraformaldehyde (PFA) solution for a duration of 10 minutes. Subsequently, they were rendered permeable by treating them with 0.1% saponin (Sigma-Aldrich) in 1× PBS for a period of 1 h at room temperature. The cells were then subjected to a blocking step employing 5% bovine serum albumin (BSA) in 1× PBS, spanning an h. This was followed by an overnight incubation with the primary antibody in a 4 °C incubator. The subsequent day, the cells underwent a triple-wash procedure using 1× PBS buffer and were subsequently exposed to the secondary antibody for a duration of 1 h at room temperature within a dark environment. For the terminal deoxynucleotidyl transferase biotin-dUTP nick end labeling (TUNEL) assay, the labeling buffer containing the enzyme was combined with the secondary antibody as per the instructions laid out in the TUNEL kit manual. Ultimately, the cells were subjected to another three rounds of washing using 1× PBS and were subsequently mounted using a DAPI solution. The acquisition of fluorescent images was carried out using a Zeiss ZEN confocal microscope equipped with an oil immersion objective.

### RNA extraction

Total RNAs from cells were extracted and purified by miRNeasy mini kit or RNeasy Mini Kit (Qiagen) according to the manuals. Total RNA concentration was evaluated by Nanodrop 2000. Fresh total RNAs were used for RNA-seq or RT-qPCR.

### RT-qPCR

CDNAs were synthesized by using 1st strand cDNA Synthesis Kit (Takara Bio), and real-time qPCR was performed by using SYBR Premix Ex Taq (Takara Bio) in a Bio-Rad Real-Time PCR System according to the manufacturer’s instructions. All PCR reactions were performed in at least three biological triplicates, normalized to the internal control genes GAPDH or beta-actin, and analyzed by using the comparative 2^−ΔΔCt^ method. Primer sequences are shown in Table S1. oligonucleotides.

### Flow cytometry

Flow cytometry was carried out according to our protocol [14]. Initially, cells were fixed using 4% paraformaldehyde (PFA) for 10 min at room temperature and subsequently rendered permeable by exposure to 0.1% saponin (Sigma-Aldrich) in 1× PBS (Gibco) for 1 h at room temperature. Following this, cells were subjected to an incubation with the primary antibody in a blocking buffer composed of 5% bovine serum albumin (BSA) and 0.1% saponin in 1× PBS buffer, maintained at 37 °C for 1 h. Subsequent to this incubation, cells were washed three times with 1× PBS, followed by additional incubation with the secondary antibody in the same blocking buffer at 37 °C for 1 h. In the case of TUNEL assay, the labeling buffer containing the enzyme was mixed with the secondary antibody according to the instructions provided by the TUNEL kit. Finally, the cells underwent another three rounds of washing with 1× PBS. The evaluation through flow cytometry was executed utilizing the CytoFLEX Flow Cytometer (Beckman Coulter). The acquired data were subsequently analyzed using FlowJo software (Treestar).

### RNA-seq

The RNA-seq analysis was conducted by submitting total RNAs to Novogene (China). Professionals from the Experimental Department at Novogene (China) were responsible for handling library preparation, sequencing, and the data analysis. For each sample, 1 μg of total RNA served as input material during RNA sample preparations. NEBNext^®^ Ultra^TM^ RNA Library Prep Kit for Illumina^®^ (NEB) was employed to generate strand-specific libraries, following the manufacturer’s guidelines. Index codes were incorporated to assign sequences to individual samples. The process involved depleting ribosomal RNA from total RNA and subsequently fragmenting the RNA using divalent cations under elevated temperature in NEBNext First-Strand Synthesis Reaction Buffer (5×). The first strand cDNA synthesis employed random hexamer primers and M-MuLV Reverse Transcriptase (RNase H minus). Subsequent second-strand cDNA synthesis utilized DNA Polymerase I and RNase H, incorporating dUTP instead of dTTP. The remaining overhangs were rendered into blunt ends through exonuclease/polymerase activities. After adenylation of 3’ ends of DNA fragments, NEBNext Adaptors with hairpin loop structures were ligated for hybridization. To select cDNA fragments of 150–200 bp length preference, library fragments underwent purification using the AMPure XP system (Beckman Coulter). The USER Enzyme (NEB) was employed to digest the second strand, involving size-selected, adaptor-ligated cDNA, at 37 °C for 15 min, followed by 5 min at 95 °C before PCR. PCR was executed using Phusion High-Fidelity DNA polymerase, Universal PCR primers, and Index Primers. Subsequently, PCR products were purified using the AMPure XP system, and the quality of the library was assessed using the Agilent Bioanalyzer 2100 system. Clustering of the index-coded samples was carried out on a cBot Cluster Generation System using TruSeq PE Cluster Kit v3-cBot-HS (Illumina), adhering to the manufacturer’s instructions. After cluster generation, the library preparations were subjected to sequencing on a Novaseq 6000 platform, resulting in the generation of 150 bp strand-specific paired-end reads.

### RNA-seq data analysis

Technicians within the Experimental Department at Novogene (China) undertook the analysis of the RNA-seq data. The initial processing involved the utilization of in-house perl scripts to manipulate the raw data, which were in fastq format. This step led to the acquisition of clean data (clean reads) by removing reads containing adapters, poly-N sequences, or those with inadequate quality from the raw data. Simultaneously, metrics such as Q20, Q30, and GC content were computed for the clean data. All subsequent analyses were conducted using the high-quality clean data. The reference genome’s index was established using hisat2-2.0.4, following which paired-end clean reads were aligned to this reference genome. To capture junction reads, STAR was employed, generating the corresponding mapping outcomes. Read counts mapped to each gene were determined using Stringtie-1.3.3b. Subsequently, the FPKM (fragments per kilobase of transcript per million mapped reads) value for each gene was computed, taking into account both the gene’s length and the mapped read count. Differential expression analysis was carried out using the edgeR_3.24.3 package. For the RNA-seq experiments, a total of three biological replicates were conducted. Genes exhibiting an adjusted p-value of less than 0.05 were identified as differentially expressed genes (DEGs).

### Assay for transposase-accessible chromatin with high-throughput sequencing (ATAC-seq)

Technicians in the Experimental Department at Novogene (China) conducted all experiments and data analyses for ATAC-seq. ATAC-seq followed established protocols [15, 16]. Initially, nuclei were isolated from the samples, and the nuclei pellet was then suspended in the Tn5 transposase reaction mixture. The transposition reaction was incubated at 37°C for 30 min. Following transposition, Equimolar Adapter1 and Adapter 2 were added, and a PCR amplification step was executed to enrich the library. Post-PCR, library purification was performed using AMPure beads, and the quality of the libraries was assessed using Qubit measurements. To prepare the indexed samples for clustering, the cBot Cluster Generation System was employed with the TruSeq PE Cluster Kit v3-cBot-HS (Illumina) in accordance with the manufacturer’s instructions. After cluster generation, the library preparations underwent sequencing on an Illumina Hiseq platform, generating 150 bp paired-end reads. Subsequently, skewer (0.2.2) was employed to remove the initial Nextera adaptor sequences from the reads. These processed reads were then aligned to a reference genome using BWA with standard parameters. Subsequently, reads were filtered to retain high-quality, non-mitochondrial chromosome, and properly paired reads (longer than 18 nt) with a minimum MAPQ score of 13. Peak calling analysis was conducted using macs2 with the following parameters: ‘macs2 callpeak –nomodel –keepdup all –call-summits’. For simulations of peaks called per input read, the aligned and de-duplicated BAM files were used without additional filtering. In the case of ATAC-seq, two biological replicates were carried out.

### Untargeted metabolomics

Technicians within Novogene’s Experimental Department (China) conducted all experimental procedures and data analysis for untargeted metabolomics. In the initial step, live cell samples were placed into EP tubes and resuspended using prechilled 80% methanol, employing vortexing. Subsequently, the samples were cooled on ice and agitated for 30 s. A sonication step of 6 min was applied to the samples, followed by centrifugation at 5000 rpm and 4 °C for 1 min. The resulting supernatant underwent freeze-drying and was then dissolved with 10% methanol. This solution was eventually introduced into the LC-MS/MS system. The LC-MS/MS analyses were carried out using a Vanquish UHPLC system (Thermo Fisher, Germany) coupled with an Orbitrap Q Exactive TMHF-X mass spectrometer (Thermo Fisher, Germany) at Novogene Co., Ltd. (Beijing, China). Samples were injected into a Hypesil Gold column (100×2.1 mm, 1.9μm) using a 17 min linear gradient at a flow rate of 0.2 mL per minute. For the positive polarity mode, eluent A (0.1% FA in water) and eluent B (in methanol) were used. In the negative polarity mode, eluent A (5 mM ammonium acetate, pH=9.0) and eluent B (in methanol) were employed. The solvent gradient progressed as follows: 2% B, 1.5 mins; 2–100% B, 3 min; 100% B, 10 min; 100–2% B, 10.1 min; 2% B, 12 min. Operating in both positive and negative polarity modes, the Q Exactive TM HF-X mass spectrometer featured a spray voltage of 3.5 kV, capillary temperature of 320°C, sheath gas flow rate of 35 psi, aux gas flow rate of 10 L per minute, S-lens RF level of 60, and aux gas heater temperature of 350 °C. The UHPLC-MS/MS-generated raw data files underwent processing using Compound Discoverer 3.1 (CD3.1, Thermo Fisher) to execute tasks such as peak alignment, peak picking, and quantitation for each metabolite. Parameters included retention time tolerance (0.2 min), actual mass tolerance (5 ppm), signal intensity tolerance (30%), and signal-to-noise ratio. Following normalization of peak intensities to total spectral intensity, the data were used to predict molecular formulas based on additive ions, molecular ion peaks, and fragment ions. Subsequently, peak matching with databases (mzCloud, mzVault, MassList) facilitated accurate qualitative and relative quantitative outcomes. Statistical analyses employed R (version R-3.4.3), Python (version 2.7.6), and CentOS (CentOS release 6.6). In cases of non-normal distribution, an area normalization approach was employed to attempt normalization. The annotated metabolites were referenced against the KEGG database, HMDB database, and LIPIDMaps database. MetaX was used for principal components analysis (PCA) and partial least squares discriminant analysis (PLS-DA). Univariate analysis (*t*-test) determined statistical significance (p value). Metabolites with VIP > 1, p-value < 0.05, and fold change ≥ 2 or ≤ 0.5 were deemed differential metabolites. Volcano plots, based on log2(Fold Change) and -log10(p value), were generated using ggplot2 in R. For clustering heat maps, z-scores of intensity areas for differential metabolites were employed for normalization and plotted using the heatmap package in R. Correlations between differential metabolites were analyzed in R, with statistically significant correlations calculated via cor.mtest and visualized using the corrplot package. Functional and pathway analyses were executed using the KEGG database. Metabolic pathway enrichment of differential metabolites was performed, considering enrichment when the ratio x/n > y/N was met, and statistical significance when p value of a metabolic pathway was < 0.05. In the context of untargeted metabolomics of N and P hiPSCs, three biological replicates were conducted.

### Chromatin immunoprecipitation (ChIP)–qPCR

The ChIP procedure involved utilizing the truChIP^®^ Chromatin Shearing Kit (Covaris) and Magna ChIP^™^ A - Chromatin Immunoprecipitation Kit (Sigma-Aldrich), following the respective manuals for guidance. To initiate chromatin shearing, cells cultured in 10 cm plates were subjected to the truChIP^®^ Chromatin Shearing Kit. The process began with washing cells using cold 1× PBS, followed by cross-linking with formaldehyde at room temperature for 5 min. To halt cross-linking, quenching buffer was added for 5 min. Cross-linked cells were separated using Corning^®^ cell scrapers and washed with cold PBS three times. Each wash involved centrifugation at 1000 g and 4 °C for 5 min. Subsequently, cells were suspended in freshly prepared chromatin shearing buffer and sonicated to achieve fragments of approximately 200 bp length, using a Covaris ME220 Focused-ultrasonicator. The immunoprecipitation (IP) of sonicated samples was conducted using the Magna ChIP™ A - Chromatin Immunoprecipitation Kit (Millipore), following the instructions provided in the kit manual. The ChIP–qPCR data were obtained from no fewer than three biological replicates. Primer sequences used in the experiments are detailed in Table S1, which is accessible in the supplementary material.

### Functional enrichment analyses

For RNA-seq, ATAC-seq, and scRNA-seq, enrichment analyses were implemented by the clusterProfiler R package or THE GENE ONTOLOGY RESOURCE (http://geneontology.org/). Terms with p value less than 0.05 were considered to be significant terms. Signaling pathway analyses were performed by large-scale molecular datasets (http://www.genome.jp/kegg/), Reactome database (https://reactome.org/) or Ingenuity Pathway Analysis (QIAGEN). ClusterProfiler R package was also used to test the statistical enrichment of DEGs in KEGG pathways by Experimental Department in Novogene (China).

### Quantification and statistical analysis

Data were represented as mean ± SD of biological replicate experiments; individual data points were also shown. The statistical significance was evaluated by using Student’s unpaired t-test (two-tailed) (comparison between two groups). One-way ANOVA was used for comparisons of three or more groups. P value less than 0.05 was considered statistically significant.

## Results

### Impact of extra copy of chromosome 21 (exCh21) on transcriptome disruption and DNA damage

**Fig. 1:**
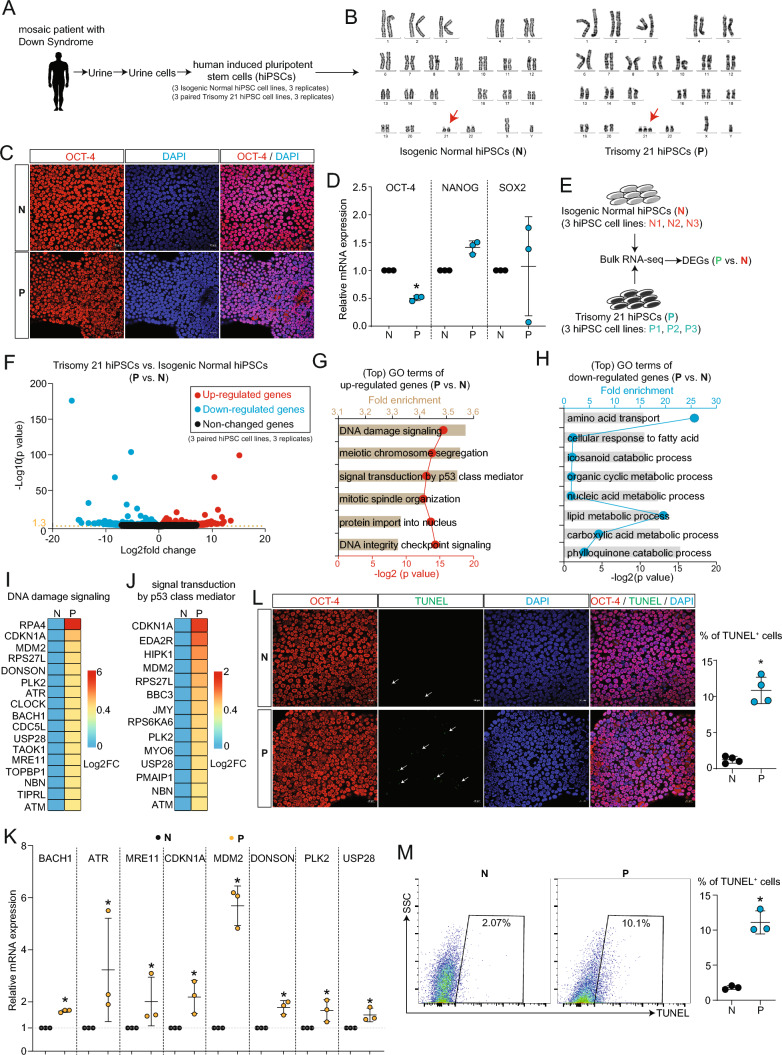
Impact of extra chromosome 21 (exCh21) on transcriptome disruption and DNA damage. **A** Outline of sample collection and hiPSCs reprogramming. Three normal hiPSC lines (isogenic controls) and three trisomy 21 hiPSC lines were generated and used in this study. **B** Karyotype analyses of hiPSC lines generated from mosaic DS patient. Red arrows show chromosome 21. P were hiPSCs with three copies of chromosome 21. N were hiPSCs with normal karyotype, as the isogenic control pairs with same genetic background. **C** Immunostaining of N and P hiPSCs. Red showed OCT4 staining. Blue (DAPI) showed nuclei staining. Scale bar, 20 µm. **D** RT-qPCR showing mRNA expression levels of pluripotency markers (OCT-4, NANOG, SOX2) in N and P hiPSCs. p*<0.05 (P vs. N). **E** Bulk RNA-seq analysis of N and P hiPSCs. Three biological replicates were performed. DEGs, differentially expressed genes. **F** Volcano plot showing differentially expressed genes (P vs. N). **G** Gene Ontology (GO) enrichment analysis of upregulated genes (P vs. N). **H** GO enrichment analysis of downregulated genes (P vs. N). **I** Heatmap showing expression levels of genes inducing DNA damage. FC, fold change (P vs. N). **J** Heatmap showing expression levels of genes inducing p53 apoptotic signaling. FC, fold change (P vs. N). **K** RT-qPCR showing expression levels of genes inducing DNA damage. p*<0.05 (P vs. N). **L** Immunostaining of TUNEL in N and P hiPSCs. Red showed OCT4 staining. Blue (DAPI) showed nuclei. Green showed TUNEL signals. Arrows showed representative TUNEL signals. Scale bar, 20 µm. Percentage (%) of TUNEL positive (TUNEL^+^) hiPSCs was quantified.p*<0.05. **M** Flow cytometry analysis of TUNEL positive (TUNEL^+^) cells in N and P hiPSCs. Percentage (%) of TUNEL^+^ hiPSCs was quantified. p*<0.05

To study the pathogenesis of Down syndrome (DS) in human, we generated human-induced pluripotent stem cells (hiPSCs) by reprogramming urine live cells isolated from DS patient (Fig. 1A–D). Karyotype analyses demonstrated mosaic karyotypes among the hiPSC lines, with some displaying normal a karyotype (N hiPSC lines) and others harboring an extra copy of chromosome 21 (exCh21) (P hiPSC lines) (Fig. 1B). This approach established an *in vitro* isogenic DS model with a consistent genetic background.

To assess the impact of exCh21 on hiPSCs, RNA-seq was employed on N and P hiPSCs (Fig. 1E, Fig. S1A–C). The results unveiled alterations in gene expression patterns due to exCh21 (Fig. 1F). Genes activated by exCh21 were associated with DNA damage and cell death (Fig. 1G, Fig. S1D), while those repressed by exCh21 were linked to metabolic processes such as lipid metabolism (Fig. 1H, Fig. S1E). Key genes implicated in DNA damage and P53 signaling were significantly upregulated in P hiPSCs (Fig. 1I-J, Fig. S1F). Additionally, exCh21 notably affected metabolic processes, including eicosanoid and fatty acid metabolism (Fig. S1G-H). RT-qPCR confirmed the activation of genes causing DNA damage by exCh21 (Fig. 1K). Immunostaining (Fig. 1L) and flow cytometry (Fig. 1M) both affirmed a higher percentage of TUNEL^+^ cells, which is the marker represents DNA damage, in P hiPSCs than in N hiPSCs. These findings demonstrated that exCh21 directly induced DNA damage and cell death in hiPSCs.

### Identification of signature genes in lipid metabolism driven by exCh21

**Fig. 2:**
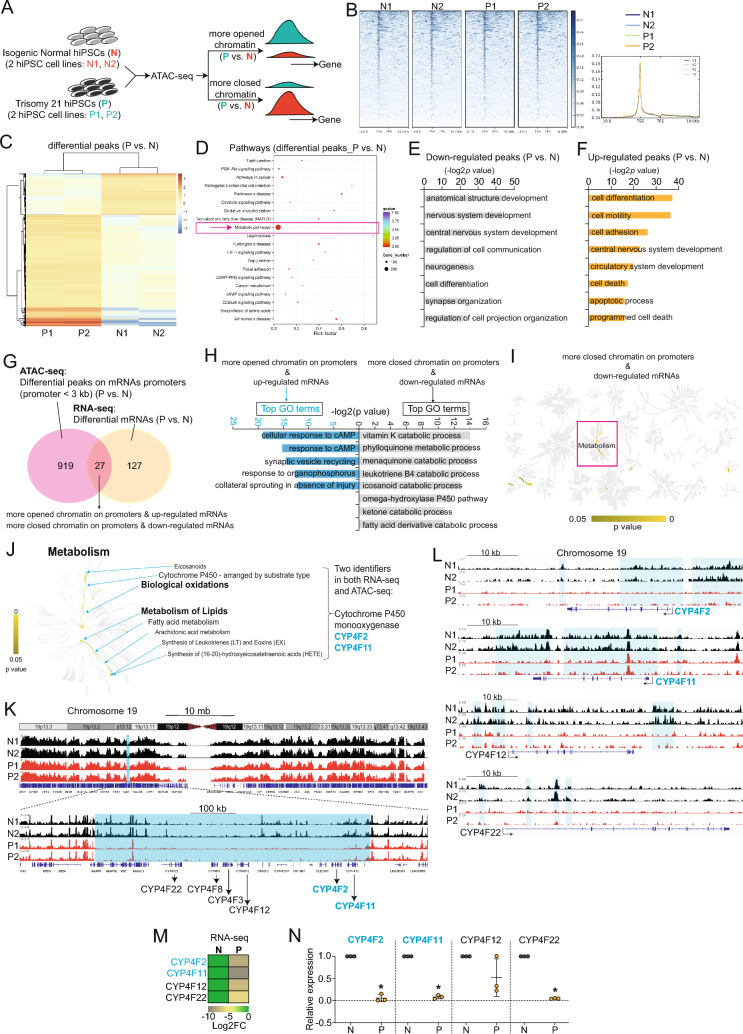
ATAC-seq reveals alterations in chromatin accessibilities induced by exCh21. **A** Schematic workflow of ATAC-seq on P and N hiPSCs. Two biological replicates were performed for ATAC-seq. N1 and N2 were two replicates of ATAC-seq on N hiPSCs. P1 and P2 were two replicates of ATAC-seq on P hiPSCs. **B** Feature distribution of ATAC-seq peaks in P and N hiPSCs. TSS, transcription start site. TES, transcription end sites. **C** Heatmap showing the differential ATAC-seq peaks (P vs. N). Scale bar, log2 (peak count). **D** KEGG pathway analyses of differential ATAC-seq peaks (P vs. N). **E** GO analysis of genes with downregulated ATAC-seq peaks (P vs. N). **F** GO analysis of genes with upregulated ATAC-seq peaks (P vs. N). **G** Overlap analysis of ATAC-seq and RNA-seq for protein-coding genes (mRNAs). **H**)GO analysis of overlapped mRNAs from (**G**). **I** Pathway analysis of overlapped mRNAs from (**G**). **J** Pathway analysis of overlapped mRNAs showing signature genes (CYP4F2, CYP4F11) involved in metabolism changes driven by exCh21. **K**-**L** Representative ATAC-seq peaks showing decreased chromatin accessibilities (P vs. N) on chromosome 19. Blue shadow boxes marked the large-scale regions with more closed chromatin accessibilities (P vs. N). **M** Heatmap showing RNA expression levels of cytochrome P450 monooxygenase family members (CYP4F2, CYP4F11) in RNA-seq. FC, fold change. **N** RT-qPCR showing RNA expression levels of cytochrome P450 monooxygenase family members (CYP4F2, CYP4F11).p*<0.05 (P vs. N)

ATAC-seq, which can define the genome-wide chromatin accessibility landscape [15, 17], was applied to P and N hiPSCs (Fig. 2A–C, Fig. S2A–E) to investigate whether exCh21 affected chromatin accessibilities and thereby elucidate the mechanism behind altered gene expression patterns observed in RNA-seq (Fig. 1). Analysis of genes with differential chromatin accessibilities (Fig. 2C) unveiled their role in crucial processes, including metabolism (Fig. 2D). Reduced chromatin accessibilities related to neural development (Fig. 2E), while increased accessibilities were linked to circulatory development and cell death (Fig. 2F). These findings suggested that exCh21 influenced gene expression within human neural and cardiovascular system, potentially through alterations in chromatin accessibilities at gene promoters [15]. This led to the identification of 27 target genes by overlapping differential ATAC-seq peaks on promoters of mRNAs with differential expression in RNA-seq (Fig. 2G). These genes exhibited either increased accessibilities and upregulation in P hiPSCs (“more opened chromatin on promoter & up-regulated mRNAs”) or decreased accessibilities and down-regulation in P hiPSCs (“more closed chromatin on promoter & down-regulated mRNAs”) (Fig. 2G). Enrichment analysis pinpointed “more closed chromatin on promoter & down-regulated mRNAs” as controlling lipid metabolism, specifically the synthesis of important bioactive metabolites (Fig. 2H-J), all known to have significant roles in human cells [18, 19]. Among these processes, two signature genes, CYP4F2 and CYP4F11, members of the cytochrome P450 monooxygenase family, were identified (Fig. 2J). Further ATAC-seq analysis showed a substantial decrease in chromatin accessibilities around CYP4F2 and CYP4F11 in P hiPSCs compared to N hiPSCs (Fig. 2K-L, Fig. S2E). Both RNA-seq (Fig. 2M) and RT-qPCR (Fig. 2N) validated the decreased RNA expression levels of CYP4F2 and CYP4F11 in P hiPSCs compared to N hiPSCs. This implicated exCh21-driven chromatin alterations in the transcriptional inactivation of CYP4F2 and CYP4F11 in P hiPSCs, possibly contributing to the observed lipid metabolism abnormalities.

### Untargeted metabolomics unveils exCh21-driven metabolome changes and potential biomarkers

**Fig. 3:**
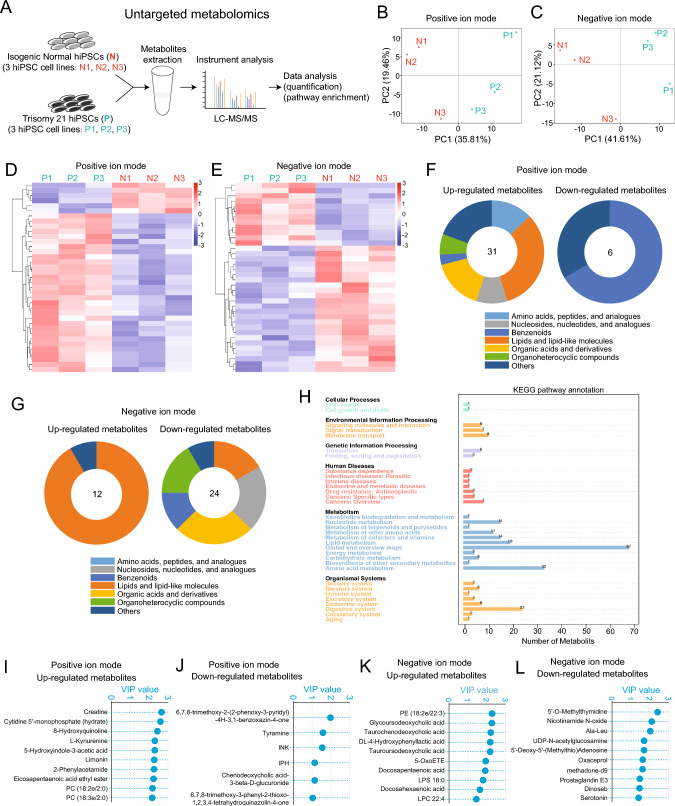
Untargeted metabolomics unveils exCh21-driven metabolome changes and potential biomarkers. **A** Schematic workflow of untargeted metabolomics in P and N hiPSCs. Three biological replicates were performed. N1, N2, and N3 were three replicates of N hiPSCs. P1, P2, and P3 were three replicates of P hiPSCs. **B**-**C** Principal component analysis (PCA) showing separation of untargeted metabolomics data in positive ion mode (B) and negative ion mode (**C**). **D**-**E** Hierarchical cluster analysis (HCA) of heatmaps showing differential metabolites (P vs. N) in positive ion mode (**D**) and negative ion mode (**E**). **F**-**G** General component analysis of differential metabolites (P vs. N) in positive ion mode (**F**) and negative ion mode (**G**). **H** KEGG pathway analysis of differential metabolites (P vs. N). **I**-**J** Variable importance in projection (VIP) scores of upregulated metabolites (P vs. N) (**I**) and downregulated metabolites (P vs. N) (**J**) in positive ion mode. **K**-**L** VIP scores of upregulated metabolites (P vs. N) (**K**) and downregulated metabolites (P vs. N) (**L**) in negative ion mode

**Fig. 4:**
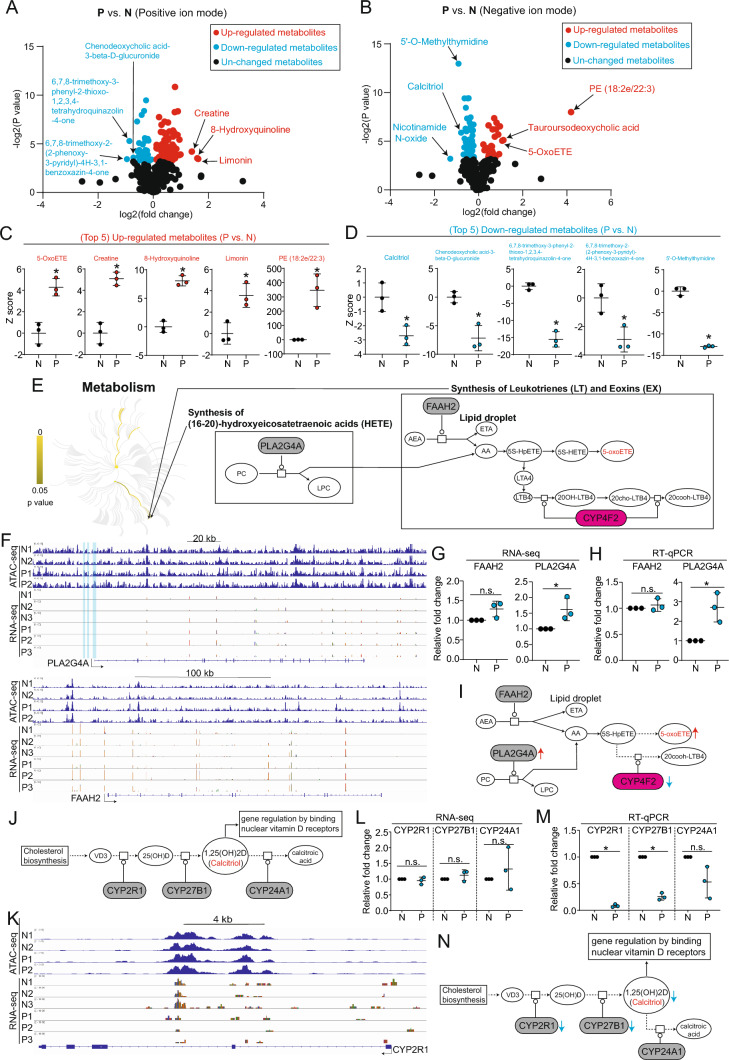
Multi-omics analyses reveal aberrant mechanisms controlling biosynthesis of 5-oxoETE and Calcitriol driven by exCh21. **A**-**B** Volcano plots showing differential metabolites (P vs. N) in positive ion mode (**A**) and negative ion mode (**B**). Metabolites with p value less than 0.05 were the differential metabolites. Arrows showed some of top differential metabolites. **C**-**D** Expression patterns of differential metabolites showing top 5 of upregulated metabolites (P vs. N) (**C**) and top 5 of downregulated metabolites (P vs. N) (**D**). p*<0.05. **E** Metabolism pathway analysis showing biosynthesis of HETE, LT, and EX, and critical synthase genes. **F** Representative peaks of ATAC-seq and RNA-seq for PLA2G4A and FAAH2 genes in N and P hiPSCs. Blue shadow boxes marked the promoter regions of PLA2G4A. Arrows showed transcriptional direction of PLA2G4A and FAAH2. **G** RNA-seq showing expression levels of FAAH2 and PLA2G4A in N and P hiPSCs (P vs. N). p*<0.05. n.s, no significance. **H** RT-qPCR showing expression levels of FAAH2 and PLA2G4A in N and P hiPSCs (P vs. N). Data were represented as mean ± SD of biological replicates; individual data points were also shown. *N* = 3, p*<0.05 (P vs. N); student t test. n.s, no significance. **I** Working model showing exCh21-driven aberrant mechanism in controlling 5-oxo-ETE biosynthesis. **J** The metabolic process of Calcitriol. VD3, vitamin D3. 25(OH)D, 25-hydroxyvitamin D. **K** Representative peaks of ATAC-seq and RNA-seq for CYP2R1 gene in N and P hiPSCs. Arrow showed transcriptional direction of CYP2R1 gene. **L** RNA-seq showing expression levels of CYP2R1, CYP27B1, and CYP24A1 in N and P hiPSCs (P vs. N). n.s, no significance. **M** RT-qPCR showing expression levels of CYP2R1, CYP27B1, and CYP24A1 in N and P hiPSCs (P vs. N). p*<0.05. **N** Working model showing exCh21-driven aberrant mechanism controlling Calcitriol bio-synthesis. VD3, vitamin D3. 25(OH)D, 25-hydroxyvitamin D

The indications that exCh21 significantly impacted metabolic processes (Fig. 2G–I) prompted the application of untargeted metabolomics to investigate how exCh21 led to metabolome changes (Fig. 3A–E, Fig. S3A-B). Many differential metabolites belong to the categories of lipids, lipid-like molecules, or organic acids and derivatives (Fig. 3F-G). Enrichment analysis emphasized the effect of exCh21 on lipid metabolism (Fig. 3H, Fig. S3C-D). The findings underscored lipids metabolism as a key event, likely stemming from the exCh21-driven downregulation of CYP4F2 and CYP4F11 (Fig. 2). Notably, the variable importance in projection (VIP) index values identified metabolite features influenced by exCh21 in hiPSCs (Fig. 3I–L). This analysis led to the identification of numerous differential metabolites and potential biomarkers (Fig. 4A–D, Fig. S4A).

### Multi-omics analysis uncovers exCh21-driven aberrant mechanisms in 5-oxo-ETE biosynthesis

Among the differential metabolites, 5-oxo-ETE emerged as one of the top upregulated metabolites (Fig. 4B-C). Integrating RNA-seq, ATAC-seq, and metabolomics data revealed the synthesis of (16–20)-hydroxyeicosatetraenoic acids (HETE), leukotrienes (LT), and eoxins (EX) as the signature event (Fig. 4E, Fig 2I), possibly due to the exCh21-driven downregulation of CYP4F2 (Fig. 2I-L, Fig. 4E). Within this synthesis process, 5-oxo-ETE was found to be a terminal metabolite in lipids metabolism (Fig. 4E). 5-oxo-ETE is a non-classic eicosanoid metabolite of arachidonic acid and the most potent naturally occurring member of the 5-HETE family of cell signaling agents. It was reported that 5-oxo-ETE stimulated various cell types, such as human leukocytes, and contributed to the development of inflammation, cancer cell growth, and other pathological and physiological events [15, 20], indicating its crucial role in humans. However, the function of 5-oxo-ETE in Down syndrome (DS) remained uncertain.

5-oxo-ETE originated from arachidonic acid 5-hydroperoxide (5S-HpETE) and arachidonic acid (AA), with AA stemming from anandamide (AEA) through fatty acid amide hydrolase 2 (FAAH2) or from arachidonic acid (ARA) via phospholipase A2 Group IVA (PLA2G4A) (Fig. 4E). Furthermore, CYP4F2 could catalyze 5S-HpETE to 20cooh-LTB4 (Fig. 4E). Mechanistically, the exCh21-downregulated CYP4F2 (Fig. 2K–N), in conjunction with PLA2G4A and FAAH2, jointly controlled 5-oxo-ETE biosynthesis. Nonetheless, the mechanism by which exCh21 led to increased 5-oxo-ETE levels remained unclear. We found enhanced chromatin accessibilities on the PLA2G4A promoter region (P vs. N) (Fig. 4F), leading to upregulated PLA2G4A expression (P vs. N) (Fig. 4F–H). In contrast, both chromatin accessibility and RNA expression levels of FAAH2 remained unchanged (Fig. 4F–H). Consequently, exCh21-driven CYP4F2 downregulation and PLA2G4A upregulation could result in AA and 5S-HpETE accumulation, culminating in elevated 5-oxo-ETE levels (Fig. 4I). Collectively, multi-omics analyses encompassing RNA-seq, ATAC-seq, and metabolomics illuminated exCh21-driven aberrant mechanisms contributing to the activation of 5-oxo-ETE biosynthesis in hiPSCs.

### Multi-omics analyses reveal exCh21-driven potential aberrant mechanism in repressing Calcitriol biosynthesis

Calcitriol, identified as one of the top downregulated metabolites in P hiPSCs (Fig. 4B, Fig. 4D), acts as the active form of vitamin D, influencing gene expression by binding to nuclear vitamin D receptors [21–24] (Fig. 4J). In pursuit of understanding its role in humans, the chromatin status and RNA expression of key genes involved in Calcitriol biosynthesis and degradation, namely CYP2R1/CYP27B1 and CYP24A1, were examined. While ATAC-seq and RNA-seq did not reveal significant changes between P and N hiPSCs (Fig. 4K-L, Fig. S4C), RT-qPCR indicated decreased RNA expression levels of both CYP2R1 and CYP27B1 in P hiPSCs compared to N hiPSCs (Fig. 4M). Conversely, CYP24A1 expression remained unchanged (Fig. 4M). Thus, the downregulation of both CYP2R1 and CYP27B1 due to exCh21 could contribute to the repression of Calcitriol biosynthesis (Fig. 4N).

### 5-oxo-ETE causes injuries

**Fig. 5:**
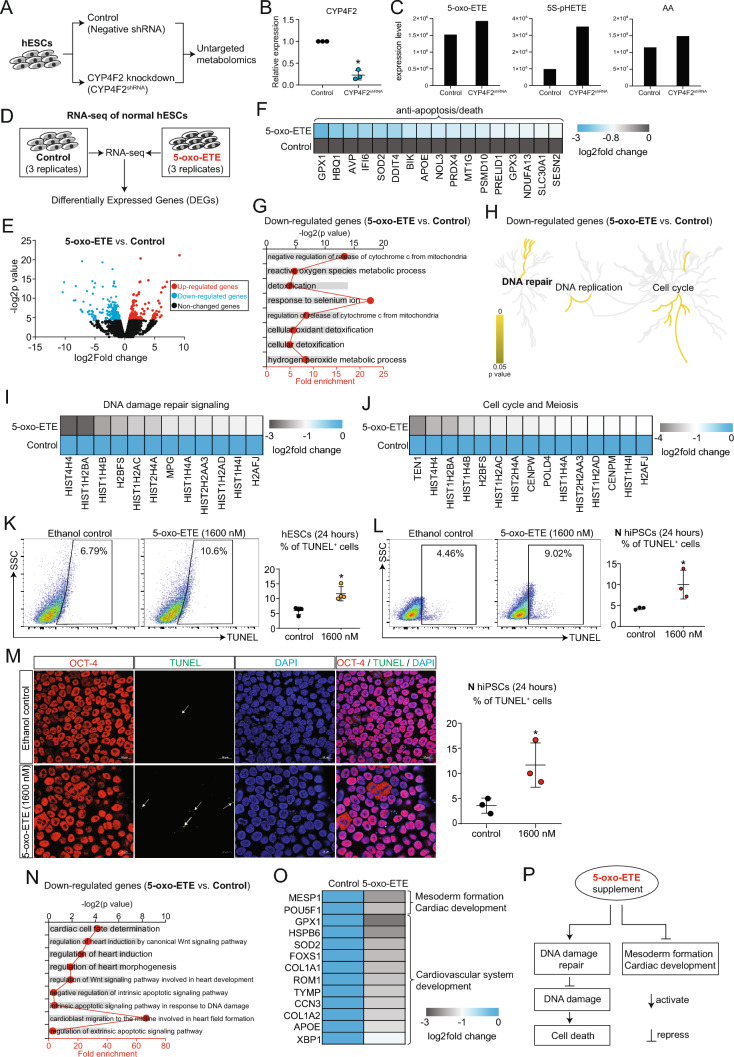
5-oxo-ETE causes injuries. **A** Scheme of untargeted metabolomics on hESCs of control (negative shRNA control) and CYP4F2 knockdown by CYP4F2 shRNA (CYP4F2^shRNA^). **B** RT-qPCR showing the expression level of CYP4F2 after shRNA knockdown. p*<0.05. **C** Untargeted metabolomics showing the expression levels of metabolites in 5-oxo-ETE biosynthesis process. **D** Bulk RNA-seq on H9 human embryonic stem cells (hESCs) treated with ethanol (Control) or 5-oxo-ETE. Final 5-oxo-ETE concentration were 1600 nM. 5-oxo-ETE was dissolved in ethanol. Control cells were treated with ethanol. Three biological replicates were applied. **E** Volcano plot showing differentially expressed genes (DEGs) (5-oxo-ETE vs. Control) in H9 hESCs. Genes with p value less than 0.05 were DEGs. **F** Heatmap showing downregulated genes induced by 5-oxo-ETE. Genes important for cell viability (anti-apoptosis) were significantly enriched. **G** GO analysis of downregulated genes (5-oxo-ETE vs. Control). **H** Signaling pathway analysis of downregulated genes (5-oxo-ETE vs. Control). Yellow color showed significant pathway terms with p value less than 0.05. **I** Heatmap showing downregulated genes induced by 5-oxo-ETE. Genes important for DNA damage repair were significantly enriched. **J** Heatmap showing downregulated genes induced by 5-oxo-ETE. Genes important for cell cycle and meiosis were significantly enriched. **K** Flow cytometry showing TUNEL^+^ cells in H9 hESCs with or without 5-oxo-ETE treatment for 24 h. Data were represented as mean ± SD of biological replicates; individual data points were also shown. *N* = 3, p*<0.05 (vs. Control); student t test. nM, nmol/L. Final 5-oxo-ETE concentration were 1600 nM. 5-oxo-ETE was dissolved in ethanol. Control cells were treated with ethanol. **L** Flow cytometry showing TUNEL^+^ cells in normal hiPSCs with or without 5-oxo-ETE treatment for 24 h. Data were represented as mean ± SD of biological replicates; individual data points were also shown. *N* = 3, p*<0.05 (vs. Control); student t test. nM, nmol/L. Final 5-oxo-ETE concentration were 1600 nM. 5-oxo-ETE was dissolved in ethanol. Control cells were treated with ethanol. **M** Immunostaining of N hiPSCs treated with or without 5-oxo-ETE treatment for 24 h. White arrows showed representative TUNEL^+^ signals. p*<0.05. **N** GO analysis of downregulated genes (5-oxo-ETE vs. Control). **O** Heatmap showing downregulated genes induced by 5-oxo-ETE. Genes important for cardiac development were significantly enriched. **P** The effects of 5-oxo-ETE supplement on hiPSCs/hESCs in vitro

ExCh21-driven downregulation of CYP4F2 (Fig. 2M-N) and upregulation of PLA2G4A facilitated the accumulation of AA and 5S-HpETE. This accumulation, in turn, potentially contributed to the buildup of 5-oxo-ETE (Fig. 4I). To confirm the role of CYP4F2 in 5-oxo-ETE accumulation, shRNA was used to knock down CYP4F2, followed by untargeted metabolomics analysis on control and CYP4F2^shRNA^ hESCs (Fig. 5A-B). The results demonstrated elevated 5-oxo-ETE levels in CYP4F2^shRNA^ hESCs compared to control hESCs, along with increased 5S-pHETE and AA (Fig. 5C). This provided evidence of the direct influence of CYP4F2 on 5-oxo-ETE biosynthesis. As 5-oxo-ETE exhibited the potential to affect human cells, RNA-seq analysis was conducted on 5-oxo-ETE-treated and control hESCs (Fig. 5D). The results indicated altered gene expression patterns (Fig. 5E, Fig. S5A) with an impact on vital molecular events, including metabolic pathways (Fig. S5B). Importantly, 5-oxo-ETE induced cell death (Fig. 5F-G, Fig. S5C), inhibited DNA damage repair and the cell cycle (Fig. 5H, Fig. S5D-E), and repressed genes involved in DNA damage repair and meiosis (Fig. 5I-J). Experimental validation confirmed an increase in the percentage of TUNEL^+^ cells in both hESCs (Fig. 5K) and normal hiPSCs (Fig. 5L-M) following 5-oxo-ETE treatment. Furthermore, RNA-seq demonstrated that 5-oxo-ETE repressed human cardiac development and heart morphogenesis within the cardiovascular system (Fig. 5N-O). Thus, 5-oxo-ETE emerged as a factor inducing injuries, including DNA damage and inhibition of mesoderm formation and cardiac development (Fig. 5P).

### Calcitriol attenuates exCh21-driven injuries

**Fig. 6:**
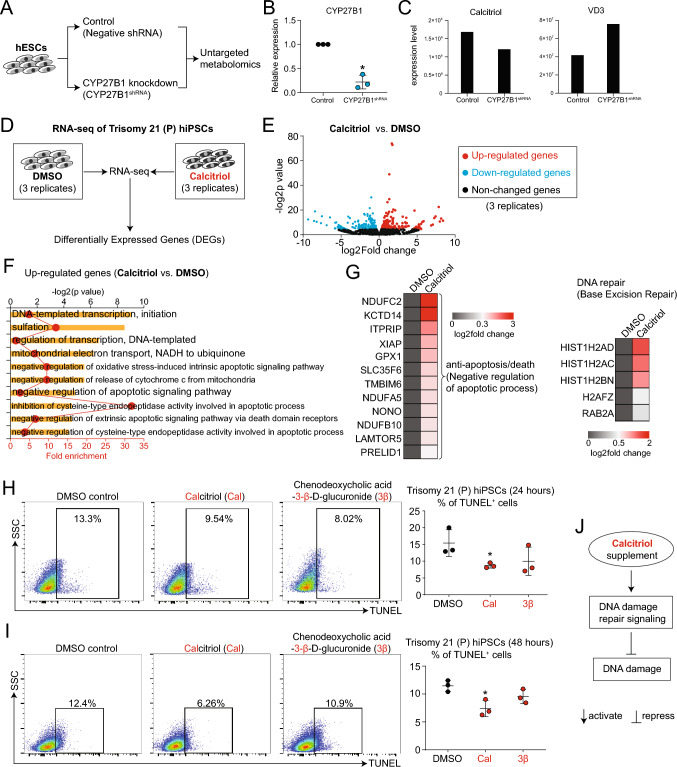
Calcitriol attenuates exCh21-driven injuries. **A** Scheme of untargeted metabolomics on hESCs of control (negative shRNA control) and CYP27B1 knockdown by CYP27B1 shRNA (CYP27B1^shRNA^). **B** RT-qPCR showing the expression level of CYP27B1. p*<0.05. **C** Untargeted metabolomics showing the levels of metabolites in Calcitriol biosynthesis process. **D** Bulk RNA-seq on P hiPSCs treated with DMSO (Control) or Calcitriol. Final concentration of Calcitriol was 25 nM. Calcitriol was dissolved in DMSO. Treatment time was 24 h. Three biological replicates were applied. **E** Volcano plot showing differentially expressed genes (DEGs) (Calcitriol vs. DMSO) on P hiPSCs. Genes with p value less than 0.05 were DEGs. **F** GO analysis of upregulated genes (Calcitriol vs. DMSO). **G** Heatmap showing upregulated genes induced by Calcitriol. Genes important for cell survival (anti-apoptosis) were significantly enriched. **H**-**I** Flow cytometry showing TUNEL^+^ cells in P hiPSCs treated with DMSO (Control), Calcitriol (Cal) or Chenodeoxycholic acid-3-β-D-glucuronide (3β) for 24 h (**H**) or 48 h (**I**). p*<0.05 (Cal vs. DMSO; 3β vs. DMSO). nM, nmol/L. Final concentration of Calcitriol (Cal) or chenodeoxycholic acid-3-β-D-glucuronide (3β) was 25 nM or 10 µM, respectively. Both Calcitriol (Cal) and Chenodeoxycholic acid-3-β-D-glucuronide (3β) were dissolved in DMSO. Control cells were treated with DMSO. **J** The effects of Calcitriol in preventing exCh21-driven injuries

The impaired function of CYP2R1 and CYP27B1, driven by ExCh21, was implicated in the diminished biosynthesis of Calcitriol (Fig. 4N). To substantiate this hypothesis, we used shRNA to attenuate CYP27B1 expression, followed by untargeted metabolomics analysis on control and CYP27B1^shRNA^ hESCs (Fig. 6A-B). The results showcased a reduction in Calcitriol levels, accompanied by an elevation in VD3 (Fig. 6C), thereby underscoring the direct contribution of CYP27B1 to Calcitriol biosynthesis.

After discovering that an aberrant mechanism driven by exCh21 caused downregulation of Calcitriol (Fig. 4N, Fig. S4A-B, Fig. 6A-C), we aimed to investigate whether supplementing Calcitriol could protect against exCh21-induced damage. We initially applied RNA-seq on Calcitriol-treated and control P hiPSCs (Fig. 6D). We observed that Calcitriol supplementation altered gene expression patterns (Fig. 6E, Fig. S6A), leading to consequential changes in signaling pathways (Fig. S6B). A pivotal outcome was the repression of cell death associated pathways by Calcitriol (Fig. 6F-G, Fig. S6C-D), coupled with the activation of DNA repair signaling (Fig. 6G, Fig. S6C). The protective role of Calcitriol was validated through experimental confirmation, wherein Calcitriol-treated P hiPSCs exhibited a markedly reduced proportion of TUNEL^+^ cells compared to control cells (Fig. 6H-I). In contrast, the control substance, chenodeoxycholic acid-3-β-D-glucuronide (3β), had no discernible effect on DNA damage in P hiPSCs (Fig. 6H-I). The culmination of evidence unequivocally established Calcitriol supplementation as a potential mechanism to counteract ExCh21-induced injuries (Fig. 6J).

### The function of 5-oxo-ETE and Calcitriol in human cardiac development

**Fig. 7:**
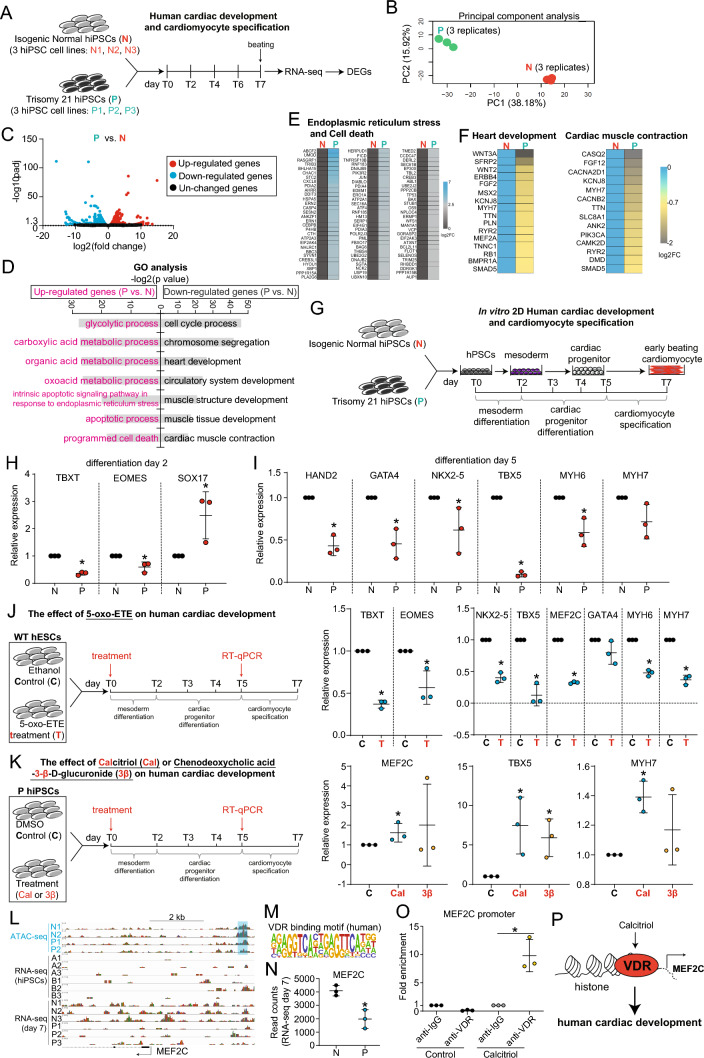
The function of 5-oxo-ETE and Calcitriol in human cardiac development. **A** Schematic workflow of human cardiac development followed with bulk RNA-seq. Three replicates of RNA-seq were performed. DEGs, differentially expressed genes. T0, T2, T4, T6 and T7 represented different stage of in vitro cardiac development on day 0, day 2, day 4, day 6, and day 7, respectively. Cells on day 7 were collected for RNA-seq. Three biological replicates were applied for RNA-seq. **B** Principal component analysis of RNA-seq datasets. **C** Volcano plot showing differentially expressed genes (DEGs). Genes with p value less than 0.05 were DEGs. **D** GO analysis showing significant enriched items for both upregulated and downregulated genes (P vs. N). **E**-**F** Heatmaps showing expression levels of genes promoting cell death (**E**), heart development, and cardiac muscle contraction (**F**). **G** 2D *in vitro* human cardiac development model for N and P hiPSCs. Samples on day 2 and day 5 were collected for RT-qPCR. **H** RT-qPCR showing RNA expression levels of mesoderm markers (TBXT, EOMES) and endoderm marker (SOX17). p*<0.05 (P vs. N). **I** RT-qPCR showing RNA expression levels of cardiogenic transcription factors and inducers (HAND2, GATA4, NKX2-5, TBX5) and cardiomyocyte-specific markers (MYH6, MYH7). p*<0.05 (P vs. N). **J** RT-qPCR showing the effect of 5-oxo-ETE in in vitro human cardiac development. Final concentration of 5-oxo-ETE was 1600 nM. Cells were treated with 5-oxo-ETE on day 0, and samples on day 5 were collected for RT-qPCR. Control group was treated with ethanol. p*<0.05 (T vs. C). C, control treated with ethanol. T, treatment with 5-oxo-ETE. **K** RT-qPCR showing the effect of Calcitriol (Cal) or chenodeoxycholic acid-3-β-D-glucuronide (3β) in *in vitro* human cardiac development. Final concentration of calcitriol (Cal) or chenodeoxycholic acid-3-β-D-glucuronide (3β) was 25 nM or 10 µM, respectively. Cells were treated with chemicals on day 0, and samples on day 5 were collected for RT-qPCR. Control group was treated with DMSO. p*<0.05 (Cal vs. C; 3β vs. C). C, control treated with DMSO. Cal, treatment with calcitriol. 3β, treatment with chenodeoxycholic acid-3-β-D-glucuronide (3β). **L** Representative ATAC-seq and RNA-seq peaks on promoter region of myogenic transcription factor MEF2C. Blue shadow boxes marked the promoter region of MEF2C, in which there were differential ATAC-seq peaks. Arrows showed transcriptional direction of MEF2C. **M** Human VDR binding motif enriched on differential ATAC-seq peaks (P vs. N) on MEF2C promoter region. **N** RNA-seq showing MEF2C expression level in N and P cells on day 7 of cardiac differentiation. p*<0.05. **O** ChIP-qPCR showing VDR binding on MEF2C promoter region in H9 hESCs. p*<0.05. Control group was treated with ethanol. Calcitriol group was treated with 5-oxo-ETE for 24 h. Final concentration of calcitriol was 25 nM. **P** The working model showing the effect of Calcitriol-VDR signaling on human cardiac development

The preceding data indicated that 5-oxo-ETE exerted a suppressive influence on genes integral to mesoderm formation, heart morphogenesis, and cardiac development (Fig. 5N–P). The prevalence of congenital heart disease (CHD) in individuals with Down syndrome (DS) [25] provided additional context to support the proposition that ExCh21-driven 5-oxo-ETE posed adverse effects on human cardiac development. Seeking further validation, we embarked on a study that involved differentiating N and P hiPSCs toward mesoderm, cardiac progenitor cells, and cardiomyocytes, followed by bulk RNA-seq analysis at day 7 (T7) (Fig. 7A–C, Fig. S7A-B). The upregulated genes in P cells primarily pertained to metabolism and cell death pathways, while the downregulated genes predominantly governed cardiac development and cardiomyocyte function (Fig. 7D-F, Fig. S7C-D). Particularly, noteworthy was the substantial repression of key transcription factors (TFs) associated with cardiogenesis and cardiomyocyte structure in P cells (Fig. 7G–I). RT-qPCR confirmed that exCh21-driven 5-oxo-ETE indeed repressed expression levels of mesodermal TFs (TBXT, EOMES), cardiogenic TFs (HAND2, GATA4, NKX2-5, TBX5), and cardiomyocyte contraction markers (MYH6, MYH7) (Fig. 7J). This provided compelling evidence for the direct impairment of human cardiac development by ExCh21.

The role of Calcitriol in this context yielded distinct observations. Calcitriol supplementation in P cells prevented the downregulation of cardiogenic genes (MEF2C, TBX5) (Fig. 7K), although the precise underlying mechanisms remained enigmatic. Intriguingly, we identified a binding motif for the vitamin D receptor (VDR) on the promoter region of myogenic transcription factor MEF2C (Fig. 7L-M). Given previous research suggesting that Calcitriol impacts gene expression by binding to its receptor VDR in the nucleus [21–24], this observation gained prominence. Additionally, the lower transcription levels of MEF2C in P cells (Fig. 7L, Fig. 7N), which correlated with diminished Calcitriol levels (Fig. 4B, Fig. 4D), suggested a potential mechanism. This mechanism was hypothesized to involve the interaction of Calcitriol with its receptor VDR, prompting binding to the MEF2C promoter and consequent enhancement of MEF2C transcription. This was subsequently confirmed through ChIP-qPCR analysis, demonstrating the promotion of VDR binding to the MEF2C promoter by Calcitriol (Fig. 7O), leading to elevated MEF2C transcription (Fig. 7K-L, Fig. 7N, Fig. 7P). Our finding highlights Calcitriol’s potential in mitigating the detrimental effects of ExCh21 on cardiac development.

## Discussion

In this study, we established an isogenic DS model through the utilization of human-induced pluripotent stem cells (hiPSCs) derived from mosaic DS patient. Employing a multi-omics approach, we comprehensively analyzed this model, unveiling a global disruption of the transcriptome by exCh21. Moreover, alterations in metabolic pathways, shifts in chromatin accessibility, and the induction of DNA damage and cell death were identified consequences of exCh21 presence. Intriguingly, our exploration led to the discovery of potential biomarkers and the revelation of exCh21-driven aberrant mechanisms governing the biosynthesis of bioactive metabolites, specifically 5-oxo-ETE and Calcitriol. By shedding light on the intricate effects of exCh21 and elucidating the underlying mechanisms of DS, our study holds the potential to pave the way for the development of targeted therapeutic strategies aimed at ameliorating the condition for DS patients.

Deciphering the pathogenesis of Down syndrome (DS) presents challenges stemming from the lack of comprehensive models capable of fully emulating the intricacies of DS. Ethical constraints limit research on DS patients and trisomic human tissues. Although animal models have been proposed for DS pathogenesis studies [26–29], their utility is not without limitations, due to evolutionary disparities and distinct arrangements of chromosome 21 [30, 31][32]. These limitations in animal models could be overcome by utilizing patient-derived iPSCs [33]. For instance, hiPSCs derived from DS patients offer a promising avenue to comprehend DS pathogenesis [34–36]. However, the utility of hiPSCs obtained from diverse patients for investigating genetic disease mechanisms is hindered by the intricate interplay of varying genetic backgrounds across individuals. This limitation underscores the significance of isogenic hiPSC lines possessing identical genetic backgrounds, which facilitate more precise investigations into DS [37–40]. Therefore, our study employed this approach to investigate the molecular underpinnings of DS by utilizing hiPSCs derived from mosaic DS patient. This model allowed for the generation of isogenic cell lines with a consistent genetic background, effectively isolating the impact of the extra chromosome 21 (ExCh21) on cellular processes. By revealing the intricate interplay between chromatin structure and gene expression, our findings offer an understanding of the influence of exCh21 on the molecular dynamics within hiPSCs, which were not reported previously.

Our integrated analyses, combining RNA-seq and ATAC-seq techniques, unveiled perturbations in metabolic pathways governed by the cytochrome P450 monooxygenase, specifically underscored by the unique signatures of CYP4F2 and CYP4F11 genes. These revelations propose the intriguing possibility that Down syndrome (DS) is intricately linked with metabolic dysregulation. To further investigate this, we delved into untargeted metabolomics and discovered distinct alterations in metabolite profiles within hiPSCs attributable to exCh21 presence. Notably, these altered metabolite patterns could serve as potential biomarkers for diagnosing DS-a facet supported by the growing body of research that has harnessed human hiPSCs to dissect metabolic aspects of DS [41, 42]. For example, mitochondria ATP metabolism was altered in trisomic hiPSCs [41]. Energy metabolism and intracellular pH were altered in neural spheroids derived from DS hiPSCs [43]. However, despite previous reports observing altered metabolisms in DS patients [44–48], comprehensive investigations into the influence of exCh21 on these metabolic aberrations or the roles of exCh21-driven differential metabolites have remained elusive. Our research, in contrast, discerned differential metabolites elicited by exCh21 within isogenic hiPSC pairs, exposing an abnormal metabolism concerning hydroxy eicosatetraenoic acid (HETE), leukotrienes (LT), and eoxins (EX). We unraveled a disruption in the biosynthesis of 5-oxo-eicosatetraenoic acid (5-oxo-ETE) and Calcitriol. In particular, we ascertained that exCh21-induced downregulation of CYP4F2 alongside upregulation of PLA2G4A culminated in the accumulation of arachidonic acid (AA) and 5S-hydroperoxyeicosatetraenoic acid (5S-HpETE), ultimately leading to the accumulation of 5-oxo-ETE within hiPSCs. This was the first report which links 5-oxo-ETE and DS in human.

While 5-oxo-ETE’s involvement in inflammation, allergic diseases, cancer, and cardiovascular diseases has been reported [20], its precise pathophysiological role, particularly in DS, remains enigmatic. Remarkably, our evidence reinforced the notion that direct supplementation of 5-oxo-ETE to hiPSCs or human embryonic stem cells (hESCs) led to DNA damage, suggesting a contributory role of 5-oxo-ETE in DS pathogenesis. Interestingly, previous study has linked 5-oxo-ETE to conditions such as myocardial infarction and stroke [49], with elevated levels detected in the serum during acute myocardial infarction (AMI) [50]. These associations underscore the potential significance of 5-oxo-ETE in the human cardiac system. Nonetheless, the role of 5-oxo-ETE in human cardiac development and cardiogenesis within the context of DS has remained elusive. Our novel findings illuminated that the supplementation of 5-oxo-ETE repressed genes critical for mesoderm formation and cardiac development in hPSCs, unmasking a hitherto unrecognized function in the human cardiac system. Notably, our data provided strong indications that the accumulation of 5-oxo-ETE driven by exCh21 could detrimentally impact human cardiac development and cardiogenesis, thereby potentially contributing to congenital heart diseases (CHD) observed in DS patients.

An additional noteworthy observation in our study pertains to the downregulation of Calcitriol orchestrated by exCh21. Calcitriol, also known as 1,25-dihydroxycholecalciferol [1,25(OH)2D], constitutes one of active forms of vitamin D. Vitamin D status in relation to DS has garnered attention, with investigations encompassing three active vitamin D metabolites, 25-hydroxyvitamin D [(25(OH)D], 1,25-dihydroxyvitamin D [1,25(OH)2D] and 24,25 dihydroxyvitamin D [24,25(OH)2D], in children [51]. In the DS group, the average values of the three vitamin D metabolites were comparable to those of an age-matched group, which indicated that DS children might not require vitamin D prescription [51]. However, another study showed that DS patients had reduced 25(OH)D levels compared to controls, and hypovitaminosis D was very frequent in DS patients, which concluded that DS patients might need higher vitamin D supplementation [52]. Regrettably, the functional ramifications of vitamin D in the context of DS have not been thoroughly investigated within these contradictory findings. In our study, we identified that Calcitriol was significantly downregulated in P hiPSCs, which is similar with the previous finding in DS patients [52]. Mechanistically, in our study, we unraveled that exCh21 orchestrates a direct downregulation of CYP2R1 and CYP27B1, thereby precipitating a dampened biosynthesis of Calcitriol. The potential utility and roles of vitamin D metabolites in DS patients, along with their implications for human cells like hiPSCs and cardiac cells, remain an enigma. Notably, our experiments illuminated that supplementation with Calcitriol could avert DNA damage in P hiPSCs, hinting at potential benefits of vitamin D metabolites for DS patients.

Moreover, the potential cardioprotective effects of Calcitriol have remained elusive, even though our findings suggest its capacity to shield P hiPSCs. Our evidence underscores that supplementation with Calcitriol could reinvigorate cardiac development within hiPSCs, likely by safeguarding cells against DNA damage and death. Thus, we substantiate that the exCh21-induced decline in Calcitriol might contribute to the pathogenesis of DS-associated CHD. Through a comprehensive multi-omics approach, we ascertained that Calcitriol could foster gene transcription by precipitating VDR binding to cardiogenic gene promoters. These discoveries advocate for the potential therapeutic relevance of Calcitriol supplementation for DS patients.

## Conclusions

Overall, our findings illuminate the landscape of aberrant metabolic events attributed to the extra copy of chromosome 21 in DS. Importantly, this study posits 5-oxo-ETE and Calcitriol as potential therapeutic targets for DS patients and those afflicted by DS-associated CHD. In summation, our multi-omics investigation provides invaluable insights into the disrupted metabolic scenarios instigated by exCh21 in DS, fostering avenues for potential therapeutic interventions in the future.

### Supplementary Information

Below is the link to the electronic supplementary material.

Supplementary file1 (PDF 2715 KB) Figure S1 RNA-seq reveals that exCh21 alters gene expression patterns and signaling pathways. (A) Principal component analysis (PCA) of RNA-seq. Three biological replicates were performed for RNA-seq. One dot showed one replicate. Red dots were replicates of N hiPSCs. Green dots were replicates of P hiPSCs. (B) Pearson correlation analysis of RNA-seq datasets. (C) Distribution of read counts in RNA-seq from N and P hiPSCs. Red color showed percentage of reads mapped to intergenic regions. Yellow color showed percentage of reads mapped to intronic regions. Blue color showed percentage of reads mapped to exons. (D) Signaling pathway analysis of upregulated genes (P vs. N). Enrichment analysis was run by Reactome. (E) Signaling pathway analysis of downregulated genes (P vs. N). Enrichment analysis was run by Reactome. (F-H) Enrichment analyses showing detailed signaling pathways controlling DNA damage (F) and metabolism processes (G-H).

Supplementary file2 (PDF 20706 KB) Figure S2 ATAC-seq reveals that exCh21 alters chromatin accessibilities. (A) Pearson correlation analysis of different ATAC-seq datasets. Two replicates were performed for ATAC-seq. N1 and N2 were two replicates of N hiPSCs. P1 and P2 were two replicates of P hiPSCs. (B) Feature distribution of ATAC-seq peaks in P and N hiPSCs. TSS, transcription start site. TES, transcription end site. (C-E) Representative ATAC-seq peaks showing large-scale alterations of chromatin accessibilities on different chromosomes driven by exCh21.

Supplementary file3 (PDF 2026 KB) Figure S3 Untargeted metabolomics reveals metabolites alterations and signature metabolic pathways driven by exCh21 in hiPSCs. (A-B) Tops of differentially expressed metabolites (P vs. N) in positive ion mode (A) and negative ion mode (B) in hiPSCs. (C-D) Metabolism pathway enrichment analyses of differentially expressed metabolites (P vs. N) in positive ion mode (C) and negative ion mode (D) in hiPSCs.

Supplementary file4 (PDF 1800 KB) Figure S4 Untargeted metabolomics uncovers potential biomarkers driven by exCh21 in hiPSCs. (A) Receiver operating characteristic (ROC) analyses showing top 5 of potential biomarkers (significantly upregulated metabolites, P vs. N). AUC, area under the curve. (B) Receiver operating characteristic (ROC) analyses showing top 5 of potential biomarkers (significantly downregulated metabolites, P vs. N). AUC, area under the curve. (C) Representative RNA-seq and ATAC-seq peaks showing RNA expression levels and chromatin accessibilities changes of cytochrome P450 monooxygenase family members, respectively.

Supplementary file5 (PDF 3376 KB) Figure S5 RNA-seq reveals the effects of 5-oxo-ETE supplement. (A) Heatmaps showing RNA expression levels of differentially expressed genes (DEGs) (5-oxo-ETE vs. Control). The number represented biological replicates of RNA-seq. (B) KEGG signaling pathway analysis of DEGs (5-oxo-ETE vs. Control). (C-D) Reactome pathway analysis of DEGs (5-oxo-ETE vs. Control).

Supplementary file6 (PDF 4257 KB) Figure S6 RNA-seq reveals the effects of Calcitriol supplement on P hiPSCs. (A) Heatmaps showing RNA expression levels of differentially expressed genes (DEGs) (Calcitriol vs. DMSO). Number represented biological replicates of RNA-seq. (B) KEGG pathway analysis of all differentially expressed genes (DEGs) (Calcitriol vs. DMSO). (C-D) Reactome enrichment analysis of all DEGs (Calcitriol vs. DMSO).

Supplementary file7 (PDF 2787 KB) Figure S7 RNA-seq reveals the impact of exCh21 on human cardiac development. (A) Percentage of RNA-seq reads mapped on human genome. Red color showed percentage of reads mapped to intergenic regions. Yellow color showed percentage of reads mapped to intronic regions. Blue color showed percentage of reads mapped to exons. (B) Heatmap showing all DEGs between N and P cells. (C) GO analysis showing enriched GO terms of differentially expressed genes (DEGs) (P vs. N). (D) KEGG pathway analysis showing enriched pathways of differentially expressed genes (DEGs) (P vs. N).

Supplementary file8 (DOCX 22 KB)

Supplementary file9 (XLSX 12 KB)

Supplementary file10 (XLSX 2557 KB)

Supplementary file11 (XLSX 9800 KB)

Supplementary file12 (XLSX 120 KB)

Supplementary file13 (XLSX 110 KB)

Supplementary file14 (XLSX 4108 KB)

Supplementary file15 (XLSX 2497 KB)

## Data Availability

Sequencing data, including all bulk RNA-seq and ATAC-seq, have been deposited at Gene Expression Omnibus (GEO). They were deposited into GSE213408, GSE213409, GSE213410, and GSE213578 in GEO, respectively. Data of untargeted metabolomics were deposited into Metabolomics Workbench (Study ID: ST002281, Project ID: PR001461). They will be publicly available as of the date of publication.

## Abbreviations

DS: Down syndrome
exCh21: An extra copy of chromosome 21
CHD: Congenital heart diseases
VDR: Vitamin D receptor
Chr.21: Chromosome 21

## References

[1] Sherman SL, Allen EG, Bean LH, Freeman SB (2007). Epidemiology of Down syndrome. Ment Retard Dev Disabil Res Rev.

[2] Park SC, Mathews RA, Zuberbuhler JR, Rowe RD, Neches WH, Lenox CC (1977). Down syndrome with congenital heart malformation. Am J Dis Child.

[3] Kusters MA, Verstegen RH, Gemen EF, de Vries E (2009). Intrinsic defect of the immune system in children with Down syndrome: a review. Clin Exp Immunol.

[4] Lott IT, Dierssen M (2010). Cognitive deficits and associated neurological complications in individuals with Down's syndrome. Lancet Neurol.

[5] Lejeune J, Turpin R, Gautier M (1959). Mongolism; a chromosomal disease (trisomy). Bull Acad Natl Med.

[6] Antonarakis SE, Skotko BG, Rafii MS, Strydom A, Pape SE, Bianchi DW, Sherman SL, Reeves RH (2020). Down Syndrome. Nat Rev Dis Primers.

[7] Maclean GA, Menne TF, Guo G, Sanchez DJ, Park IH, Daley GQ, Orkin SH (2012). Altered hematopoiesis in trisomy 21 as revealed through in vitro differentiation of isogenic human pluripotent cells. Proc Natl Acad Sci U S A.

[8] Alford KA, Slender A, Vanes L, Li Z, Fisher EM, Nizetic D, Orkin SH, Roberts I, Tybulewicz VL (2010). Perturbed hematopoiesis in the Tc1 mouse model of Down syndrome. Blood.

[9] Meharena HS, Marco A, Dileep V, Lockshin ER, Akatsu GY, Mullahoo J, Watson LA, Ko T, Guerin LN, Abdurrob F (2022). Down-syndrome-induced senescence disrupts the nuclear architecture of neural progenitors. Cell Stem Cell.

[10] Assenza GE, Autore C, Marino B (2007). Hypertrophic cardiomyopathy in a patient with Down's syndrome. J Cardiovasc Med (Hagerstown).

[11] Mahadevaiah G, Gupta M, Ashwath R (2015). Down Syndrome with Complete Atrioventricular Septal Defect, Hypertrophic Cardiomyopathy, and Pulmonary Vein Stenosis. Tex Heart Inst J.

[12] Dimopoulos K, Constantine A, Clift P, Condliffe R, Moledina S, Jansen K, Inuzuka R, Veldtman GR, Cua CL, Tay ELW (2023). Cardiovascular Complications of Down Syndrome: Scoping Review and Expert Consensus. Circulation.

[13] Zhou T, Benda C, Duzinger S, Huang Y, Li X, Li Y, Guo X, Cao G, Chen S, Hao L (2011). Generation of induced pluripotent stem cells from urine. J Am Soc Nephrol.

[14] Liu J, Liu S, Gao H, Han L, Chu X, Sheng Y, Shou W, Wang Y, Liu Y, Wan J, Yang L (2020). Genome-wide studies reveal the essential and opposite roles of ARID1A in controlling human cardiogenesis and neurogenesis from pluripotent stem cells. Genome Biol.

[15] Buenrostro JD, Giresi PG, Zaba LC, Chang HY, Greenleaf WJ (2013). Transposition of native chromatin for fast and sensitive epigenomic profiling of open chromatin, DNA-binding proteins and nucleosome position. Nat Methods.

[16] Corces MR, Trevino AE, Hamilton EG, Greenside PG, Sinnott-Armstrong NA, Vesuna S, Satpathy AT, Rubin AJ, Montine KS, Wu B (2017). An improved ATAC-seq protocol reduces background and enables interrogation of frozen tissues. Nat Methods.

[17] Yan F, Powell DR, Curtis DJ, Wong NC (2020). From reads to insight: a hitchhiker's guide to ATAC-seq data analysis. Genome Biol.

[18] Feltenmark S, Gautam N, Brunnstrom A, Griffiths W, Backman L, Edenius C, Lindbom L, Bjorkholm M, Claesson HE (2008). Eoxins are proinflammatory arachidonic acid metabolites produced via the 15-lipoxygenase-1 pathway in human eosinophils and mast cells. Proc Natl Acad Sci U S A.

[19] Fruteau de Laclos B, Braquet P, Borgeat P (1984). Characteristics of leukotriene (LT) and hydroxy eicosatetraenoic acid (HETE) synthesis in human leukocytes in vitro: effect of arachidonic acid concentration. Prostaglandins Leukot Med.

[20] Powell WS, Rokach J (2013). The eosinophil chemoattractant 5-oxo-ETE and the OXE receptor. Prog Lipid Res.

[21] Kato S (2000). The function of vitamin D receptor in vitamin D action. J Biochem.

[22] Ozono K, Saito M, Miura D, Michigami T, Nakajima S, Ishizuka S (1999). Analysis of the molecular mechanism for the antagonistic action of a novel 1alpha,25-dihydroxyvitamin D(3) analogue toward vitamin D receptor function. J Biol Chem.

[23] Cianferotti L, Cox M, Skorija K, Demay MB (2007). Vitamin D receptor is essential for normal keratinocyte stem cell function. Proc Natl Acad Sci U S A.

[24] Kongsbak M, Levring TB, Geisler C, von Essen MR (2013). The vitamin d receptor and T cell function. Front Immunol.

[25] Godown J, Fountain D, Bansal N, Ameduri R, Anderson S, Beasley G, Burstein D, Knecht K, Molina K, Pye S (2022). Heart Transplantation in Children With Down Syndrome. J Am Heart Assoc.

[26] Scott-McKean JJ, Chang B, Hurd RE, Nusinowitz S, Schmidt C, Davisson MT, Costa AC (2010). The mouse model of Down syndrome Ts65Dn presents visual deficits as assessed by pattern visual evoked potentials. Invest Ophthalmol Vis Sci.

[27] Brault V, Pereira P, Duchon A, Herault Y (2006). Modeling chromosomes in mouse to explore the function of genes, genomic disorders, and chromosomal organization. PLoS Genet.

[28] Yu Y, Bradley A (2001). Engineering chromosomal rearrangements in mice. Nat Rev Genet.

[29] Ramirez-Solis R, Liu P, Bradley A (1995). Chromosome engineering in mice. Nature.

[30] Davisson MT, Schmidt C, Akeson EC (1990). Segmental trisomy of murine chromosome 16: a new model system for studying Down syndrome. Prog Clin Biol Res.

[31] Herault Y, Delabar JM, Fisher EMC, Tybulewicz VLJ, Yu E, Brault V (2017). Rodent models in Down syndrome research: impact and future opportunities. Dis Model Mech.

[32] Rueda N, Florez J, Martinez-Cue C (2012). Mouse models of Down syndrome as a tool to unravel the causes of mental disabilities. Neural Plast.

[33] Wen Z, Nguyen HN, Guo Z, Lalli MA, Wang X, Su Y, Kim NS, Yoon KJ, Shin J, Zhang C (2014). Synaptic dysregulation in a human iPS cell model of mental disorders. Nature.

[34] Huo HQ, Qu ZY, Yuan F, Ma L, Yao L, Xu M, Hu Y, Ji J, Bhattacharyya A, Zhang SC, Liu Y (2018). Modeling Down Syndrome with Patient iPSCs Reveals Cellular and Migration Deficits of GABAergic Neurons. Stem Cell Reports.

[35] Chen C, Jiang P, Xue H, Peterson SE, Tran HT, McCann AE, Parast MM, Li S, Pleasure DE, Laurent LC (2014). Role of astroglia in Down's syndrome revealed by patient-derived human-induced pluripotent stem cells. Nat Commun.

[36] Tang XY, Xu L, Wang J, Hong Y, Wang Y, Zhu Q, Wang D, Zhang XY, Liu CY, Fang KH (2021). DSCAM/PAK1 pathway suppression reverses neurogenesis deficits in iPSC-derived cerebral organoids from patients with Down syndrome. J Clin Invest.

[37] Soldner F, Laganiere J, Cheng AW, Hockemeyer D, Gao Q, Alagappan R, Khurana V, Golbe LI, Myers RH, Lindquist S (2011). Generation of isogenic pluripotent stem cells differing exclusively at two early onset Parkinson point mutations. Cell.

[38] Niemitz E (2014). Isogenic iPSC-derived models of disease. Nature Genetics.

[39] Murray A, Letourneau A, Canzonetta C, Stathaki E, Gimelli S, Sloan-Bena F, Abrehart R, Goh P, Lim S, Baldo C (2015). Brief report: isogenic induced pluripotent stem cell lines from an adult with mosaic down syndrome model accelerated neuronal ageing and neurodegeneration. Stem Cells.

[40] Weick JP, Held DL, Bonadurer GF, Doers ME, Liu Y, Maguire C, Clark A, Knackert JA, Molinarolo K, Musser M (2013). Deficits in human trisomy 21 iPSCs and neurons. Proc Natl Acad Sci U S A.

[41] Mollo N, Esposito M, Aurilia M, Scognamiglio R, Accarino R, Bonfiglio F, Cicatiello R, Charalambous M, Procaccini C, Micillo T (2021). Human Trisomic iPSCs from Down Syndrome Fibroblasts Manifest Mitochondrial Alterations Early during Neuronal Differentiation. Biology (Basel).

[42] Sobol M, Klar J, Laan L, Shahsavani M, Schuster J, Anneren G, Konzer A, Mi J, Bergquist J, Nordlund J (2019). Transcriptome and Proteome Profiling of Neural Induced Pluripotent Stem Cells from Individuals with Down Syndrome Disclose Dynamic Dysregulations of Key Pathways and Cellular Functions. Mol Neurobiol.

[43] Kashirina A, Gavrina A, Kryukov E, Elagin V, Kolesova Y, Artyuhov A, Momotyuk E, Abdyyev V, Meshcheryakova N, Zagaynova E (2021). Energy Metabolism and Intracellular pH Alteration in Neural Spheroids Carrying Down Syndrome. Biomedicines.

[44] Gross TJ, Doran E, Cheema AK, Head E, Lott IT, Mapstone M (2019). Plasma metabolites related to cellular energy metabolism are altered in adults with Down syndrome and Alzheimer's disease. Dev Neurobiol.

[45] Caracausi M, Ghini V, Locatelli C, Mericio M, Piovesan A, Antonaros F, Pelleri MC, Vitale L, Vacca RA, Bedetti F (2018). Plasma and urinary metabolomic profiles of Down syndrome correlate with alteration of mitochondrial metabolism. Sci Rep.

[46] Obeid R, Hartmuth K, Herrmann W, Gortner L, Rohrer TR, Geisel J, Reed MC, Nijhout HF (2012). Blood biomarkers of methylation in Down syndrome and metabolic simulations using a mathematical model. Mol Nutr Food Res.

[47] Pogribna M, Melnyk S, Pogribny I, Chango A, Yi P, James SJ (2001). Homocysteine metabolism in children with Down syndrome: in vitro modulation. Am J Hum Genet.

[48] Moreau M, Benhaddou S, Dard R, Tolu S, Hamze R, Vialard F, Movassat J, Janel N (2021). Metabolic Diseases and Down Syndrome: How Are They Linked Together?. Biomedicines.

[49] Helgadottir A, Manolescu A, Thorleifsson G, Gretarsdottir S, Jonsdottir H, Thorsteinsdottir U, Samani NJ, Gudmundsson G, Grant SF, Thorgeirsson G (2004). The gene encoding 5-lipoxygenase activating protein confers risk of myocardial infarction and stroke. Nat Genet.

[50] Lai Q, Yuan G, Shen L, Zhang L, Fu F, Liu Z, Zhang Y, Kou J, Liu S, Yu B, Li F (2021). Oxoeicosanoid receptor inhibition alleviates acute myocardial infarction through activation of BCAT1. Basic Res Cardiol.

[51] Del Arco C, Riancho JA, Luzuriaga C, Gonzalez-Macias J, Florez J (1992). Vitamin D status in children with Down's syndrome. J Intellect Disabil Res.

[52] Stagi S, Lapi E, Romano S, Bargiacchi S, Brambilla A, Giglio S, Seminara S, de Martino M (2015). Determinants of vitamin d levels in children and adolescents with down syndrome. Int J Endocrinol.

