# Supplementary figures and images for: Trisomy 21-driven metabolite alterations are linked to cellular injuries in Down syndrome
Source: Cell Mol Life Sci. 2024 Mar 3;81(1):112. doi: 10.1007/s00018-024-05127-0 (PMC10909777; doi:10.1007/s00018-024-05127-0)

Supplementary Fig 1

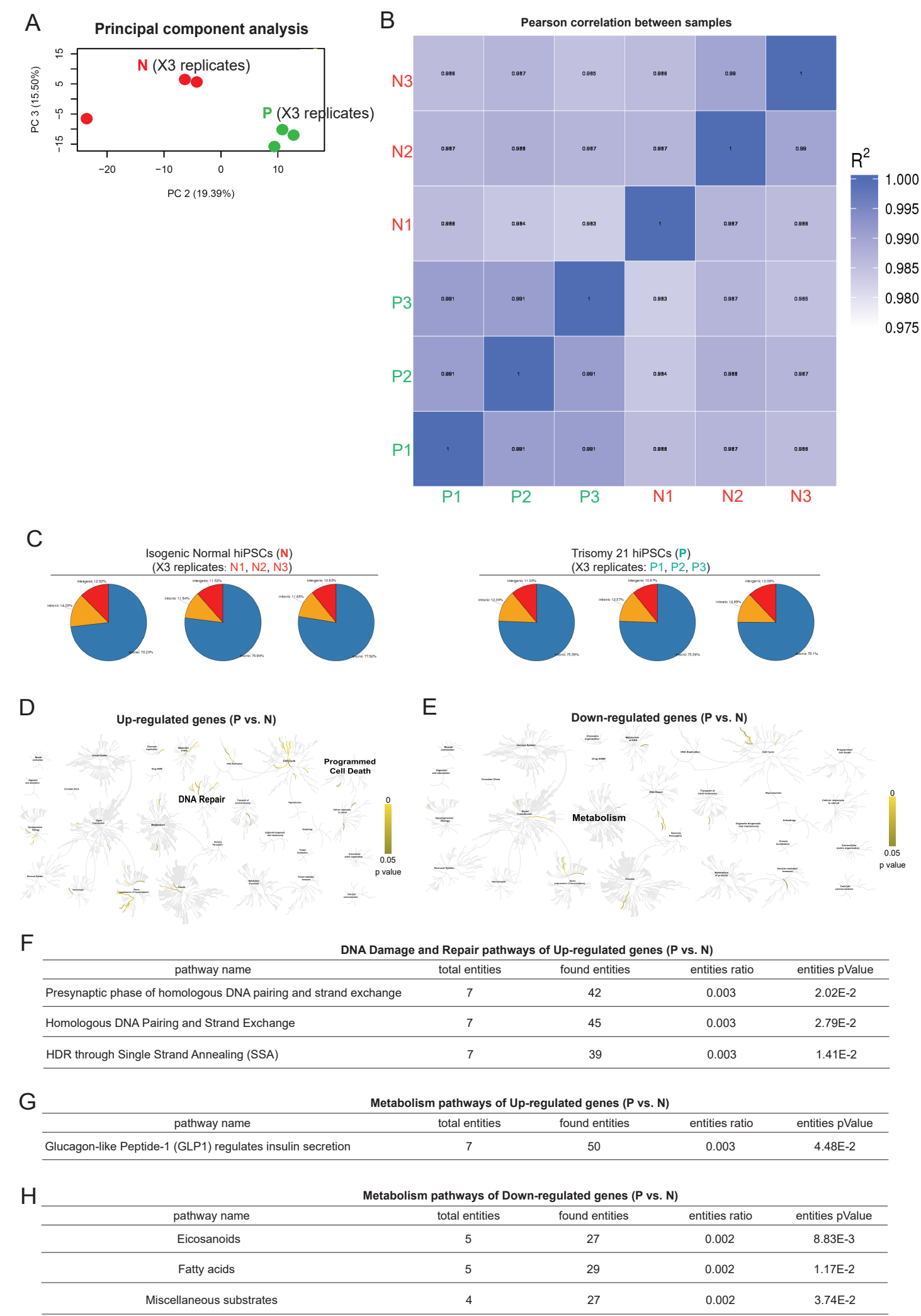

Supplement: Supplementary file 1 — Supplementary file1 (PDF 2715 KB) Figure S1 RNA-seq reveals that exCh21 alters gene expression patterns and signaling pathways. (A) Principal component analysis (PCA) of RNA-seq. Three biological replicates were performed for RNA-seq. One dot showed one replicate. Red dots were replicates of N hiPSCs. Green dots were replicates of P hiPSCs. (B) Pearson correlation analysis of RNA-seq datasets. (C) Distribution of read counts in RNA-seq from N and P hiPSCs. Red color showed percentage of reads mapped to intergenic regions. Yellow color showed percentage of reads mapped to intronic regions. Blue color showed percentage of reads mapped to exons. (D) Signaling pathway analysis of upregulated genes (P vs. N). Enrichment analysis was run by Reactome. (E) Signaling pathway analysis of downregulated genes (P vs. N). Enrichment analysis was run by Reactome. (F-H) Enrichment analyses showing detailed signaling pathways controlling DNA damage (F) and metabolism processes (G-H). [file 18_2024_5127_MOESM1_ESM.pdf]

Supplementary Fig 2

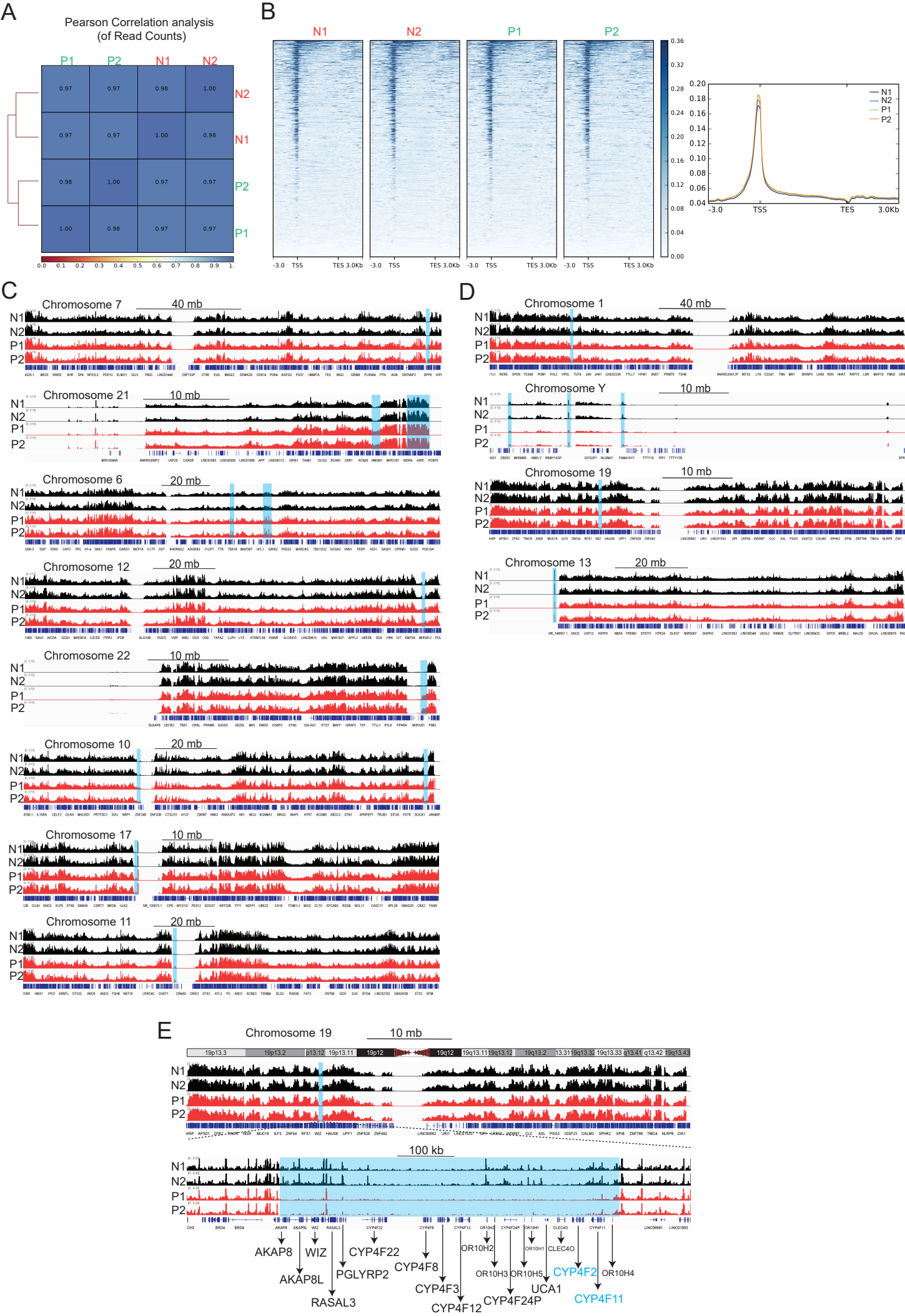

Supplement: Supplementary file 2 — Supplementary file2 (PDF 20706 KB) Figure S2 ATAC-seq reveals that exCh21 alters chromatin accessibilities. (A) Pearson correlation analysis of different ATAC-seq datasets. Two replicates were performed for ATAC-seq. N1 and N2 were two replicates of N hiPSCs. P1 and P2 were two replicates of P hiPSCs. (B) Feature distribution of ATAC-seq peaks in P and N hiPSCs. TSS, transcription start site. TES, transcription end site. (C-E) Representative ATAC-seq peaks showing large-scale alterations of chromatin accessibilities on different chromosomes driven by exCh21. [file 18_2024_5127_MOESM2_ESM.pdf]

Supplementary Fig 3

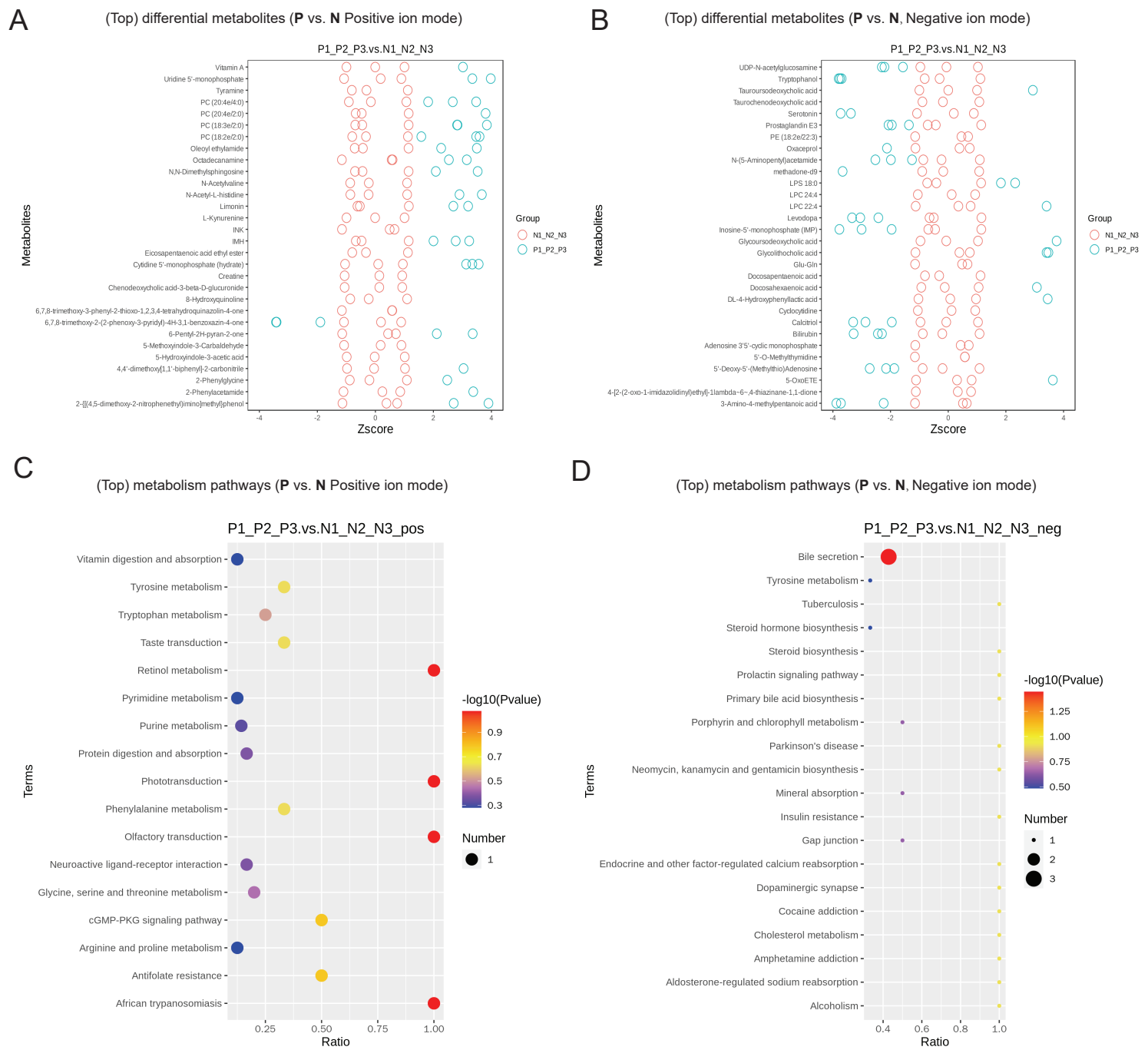

Supplement: Supplementary file 3 — Supplementary file3 (PDF 2026 KB) Figure S3 Untargeted metabolomics reveals metabolites alterations and signature metabolic pathways driven by exCh21 in hiPSCs. (A-B) Tops of differentially expressed metabolites (P vs. N) in positive ion mode (A) and negative ion mode (B) in hiPSCs. (C-D) Metabolism pathway enrichment analyses of differentially expressed metabolites (P vs. N) in positive ion mode (C) and negative ion mode (D) in hiPSCs. [file 18_2024_5127_MOESM3_ESM.pdf]

Supplementary Fig 4

A (Top 5) Up-regulated metabolites (P vs. N)

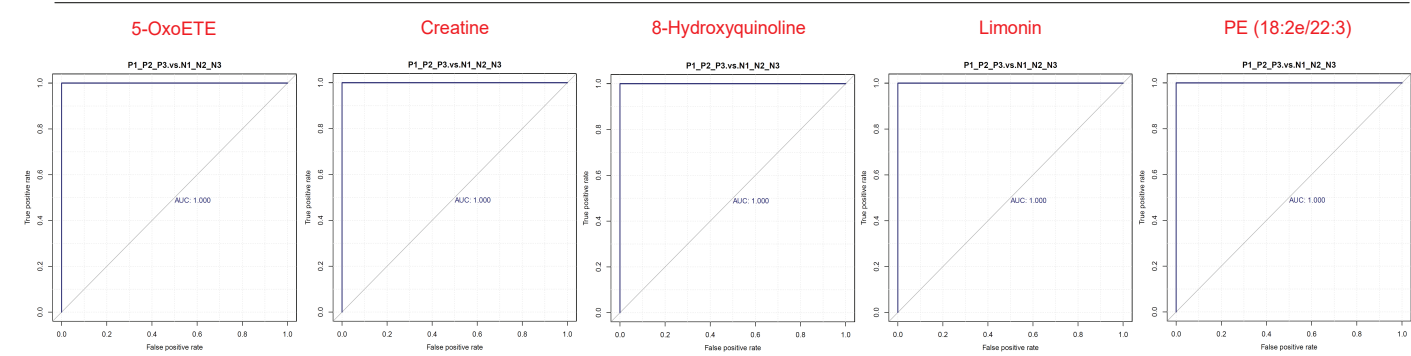

B (Top 5) Down-regulated metabolites (P vs. N)

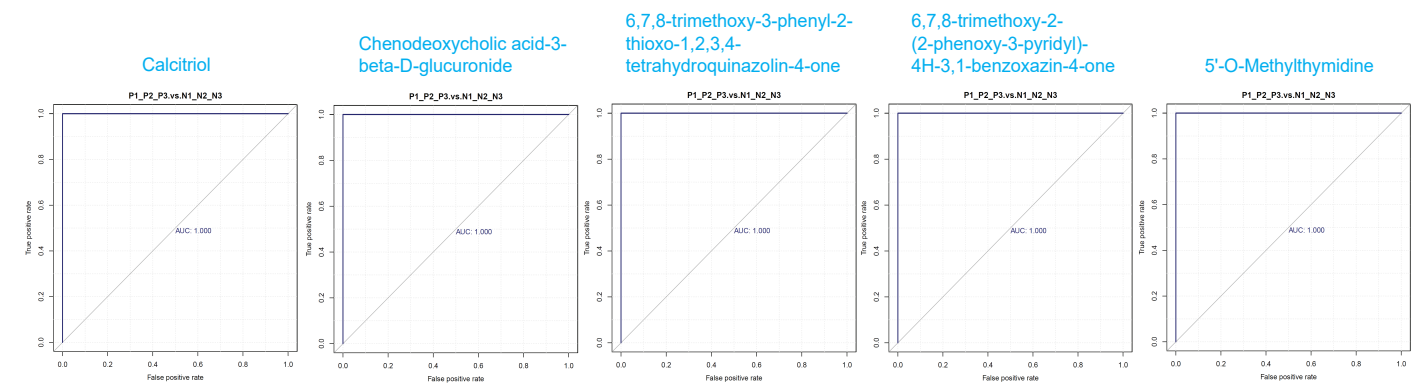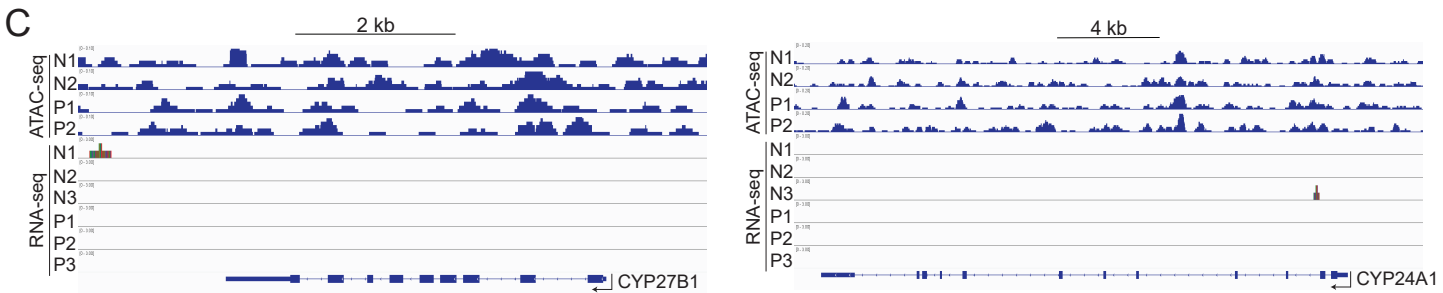

Supplement: Supplementary file 4 — Supplementary file4 (PDF 1800 KB) Figure S4 Untargeted metabolomics uncovers potential biomarkers driven by exCh21 in hiPSCs. (A) Receiver operating characteristic (ROC) analyses showing top 5 of potential biomarkers (significantly upregulated metabolites, P vs. N). AUC, area under the curve. (B) Receiver operating characteristic (ROC) analyses showing top 5 of potential biomarkers (significantly downregulated metabolites, P vs. N). AUC, area under the curve. (C) Representative RNA-seq and ATAC-seq peaks showing RNA expression levels and chromatin accessibilities changes of cytochrome P450 monooxygenase family members, respectively. [file 18_2024_5127_MOESM4_ESM.pdf]

Supplementary Fig 5

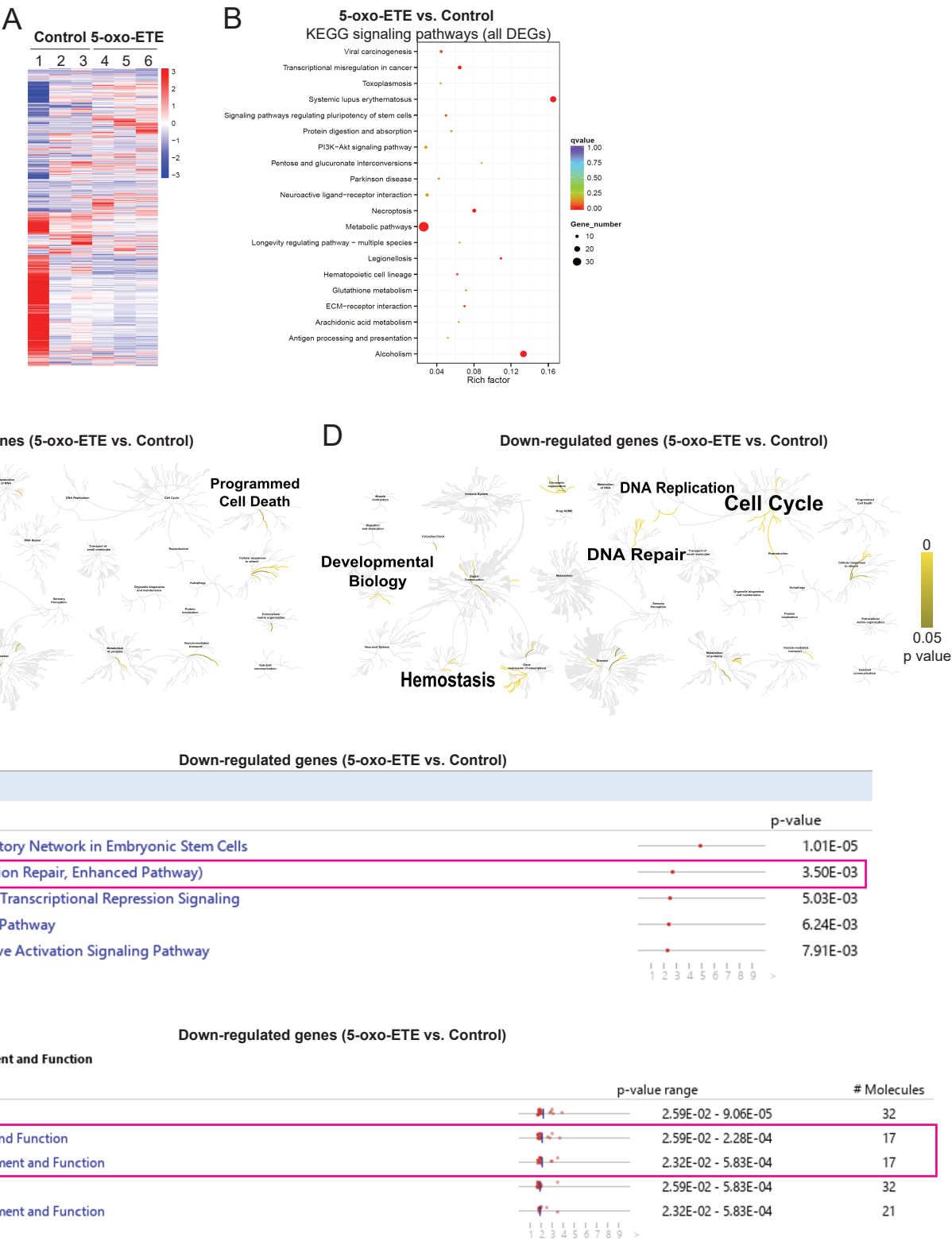

Supplement: Supplementary file 5 — Supplementary file5 (PDF 3376 KB) Figure S5 RNA-seq reveals the effects of 5-oxo-ETE supplement. (A) Heatmaps showing RNA expression levels of differentially expressed genes (DEGs) (5-oxo-ETE vs. Control). The number represented biological replicates of RNA-seq. (B) KEGG signaling pathway analysis of DEGs (5-oxo-ETE vs. Control). (C-D) Reactome pathway analysis of DEGs (5-oxo-ETE vs. Control). [file 18_2024_5127_MOESM5_ESM.pdf]

Supplementary Fig 6

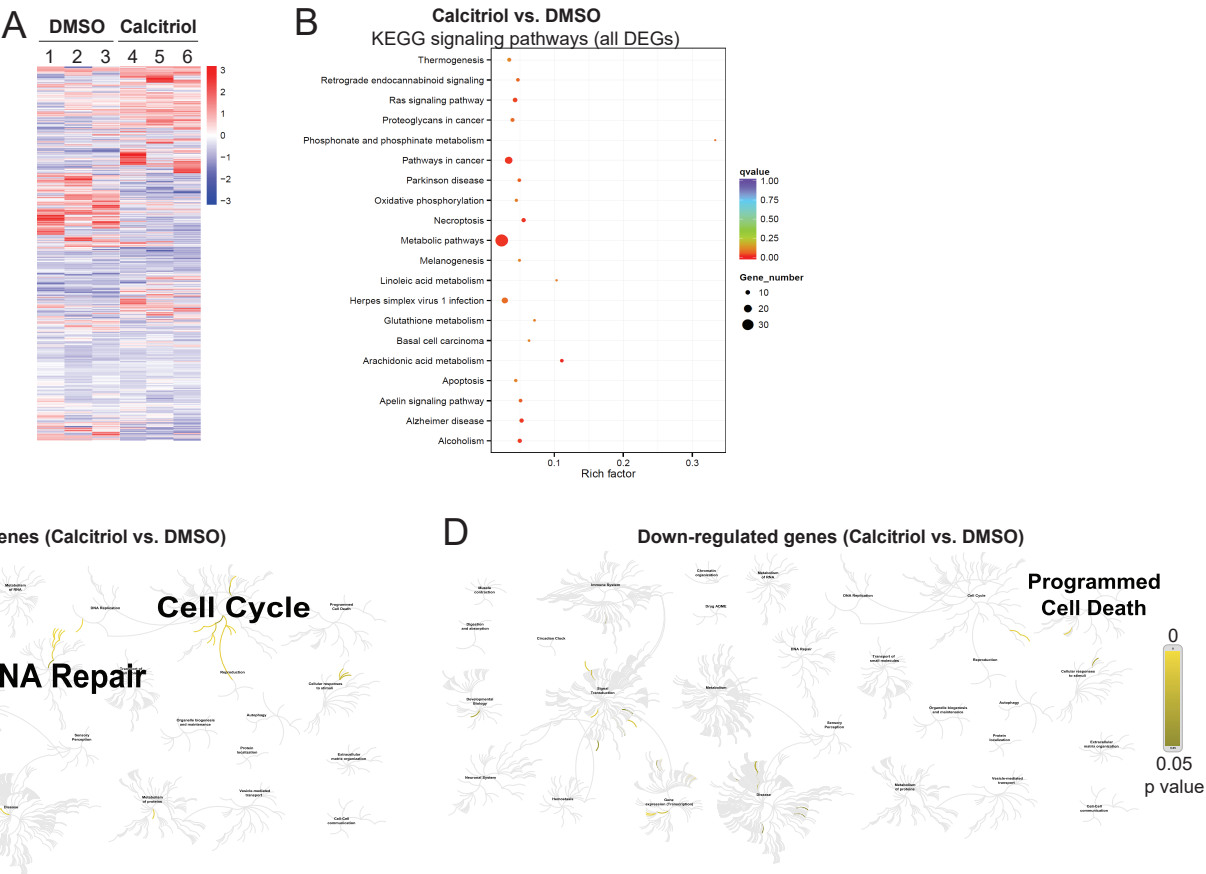

Supplement: Supplementary file 6 — Supplementary file6 (PDF 4257 KB) Figure S6 RNA-seq reveals the effects of Calcitriol supplement on P hiPSCs. (A) Heatmaps showing RNA expression levels of differentially expressed genes (DEGs) (Calcitriol vs. DMSO). Number represented biological replicates of RNA-seq. (B) KEGG pathway analysis of all differentially expressed genes (DEGs) (Calcitriol vs. DMSO). (C-D) Reactome enrichment analysis of all DEGs (Calcitriol vs. DMSO). [file 18_2024_5127_MOESM6_ESM.pdf]

Supplementary Fig 7

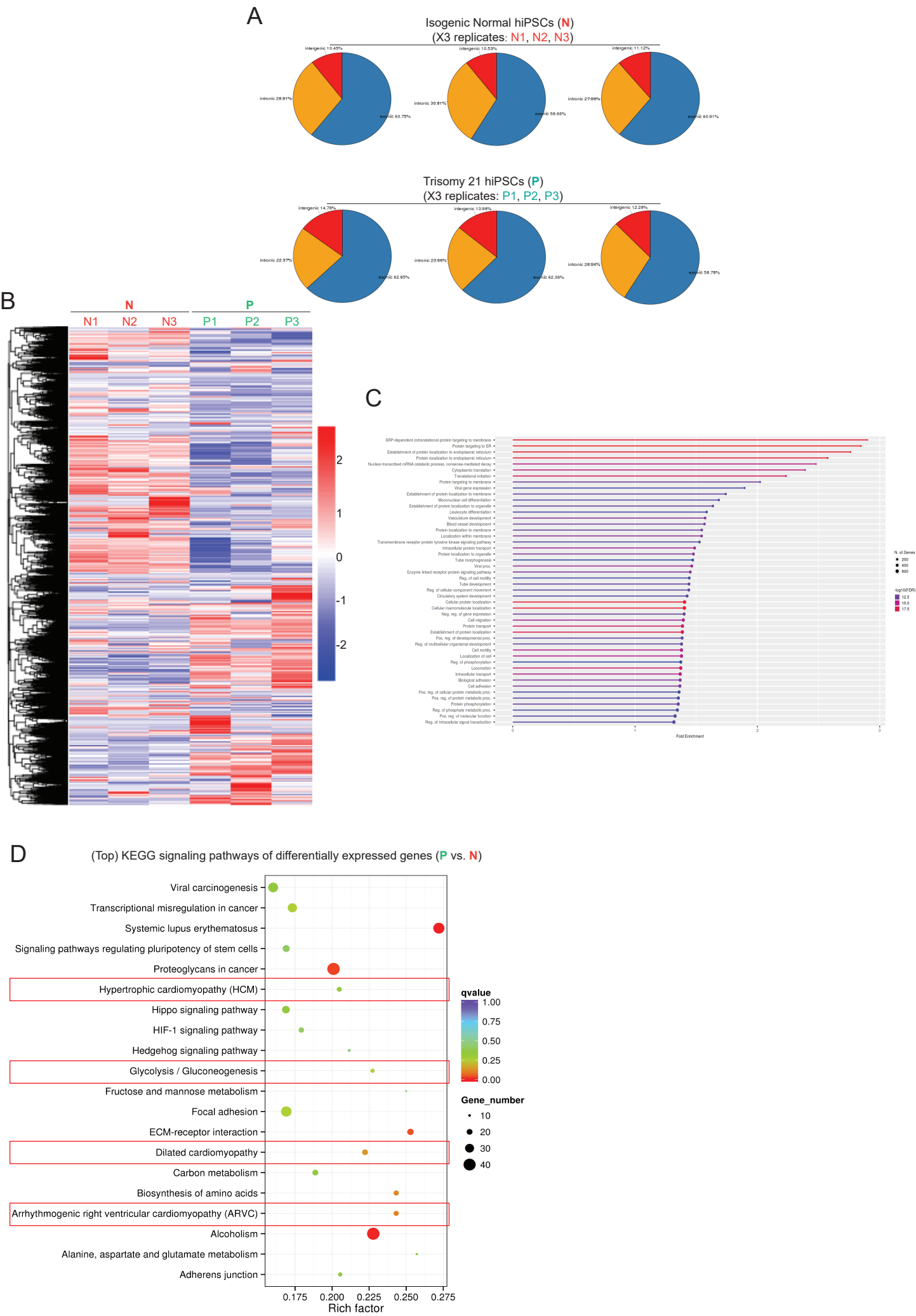

Supplement: Supplementary file 7 — Supplementary file7 (PDF 2787 KB) Figure S7 RNA-seq reveals the impact of exCh21 on human cardiac development. (A) Percentage of RNA-seq reads mapped on human genome. Red color showed percentage of reads mapped to intergenic regions. Yellow color showed percentage of reads mapped to intronic regions. Blue color showed percentage of reads mapped to exons. (B) Heatmap showing all DEGs between N and P cells. (C) GO analysis showing enriched GO terms of differentially expressed genes (DEGs) (P vs. N). (D) KEGG pathway analysis showing enriched pathways of differentially expressed genes (DEGs) (P vs. N). [file 18_2024_5127_MOESM7_ESM.pdf]
