# Supplementary material for: Trisomy 21-driven metabolite alterations are linked to cellular injuries in Down syndrome
Source: Cell Mol Life Sci. 2024 Mar 3;81(1):112. doi: 10.1007/s00018-024-05127-0 (PMC10909777; doi:10.1007/s00018-024-05127-0)
Supplement: Supplementary file 8 — Supplementary file8 (DOCX 22 KB) [file 18_2024_5127_MOESM8_ESM.docx]

**KEY RESOURCES TABLE**

| **REAGENT or RESOURCE** | **SOURCE** | **IDENTIFIER** |
| --- | --- | --- |
| **Antibodies** | | |
| Cardiac Troponin T | Thermo Fisher | Cat: MS-295-P, RRID:AB_61806 |
| Goat anti-mouse Alexa Fluor 488 | Invitrogen | Cat: A28175  RRID:AB_2534069 |
| Goat anti-rabbit Alexa Fluor 555 | Invitrogen | Cat: A27039  RRID:AB_2536100 |
| Vitamin D3 Receptor (D2K6W) Rabbit mAb | Cell Signaling Technology | Cat: 12550  RRID:AB_2637002 |
| OCT-4 | Cell Signaling Technology | Cat: #2750  RRID:AB_823583 |
| **Critical commercial reagents** | | |
| SYBR Premix Ex Taq | Takara Bio | RR420A |
| DMEM, no glucose | Gibco | 11966025 |
| 1st strand cDNA Synthesis Kit | Takara Bio | 6110A |
| mTesR1 medium | STEMCELL Technologies | 85850 |
| ReLeSR™ Passaging Reagent | STEMCELL Technologies | 100-0484 |
| Rock inhibitor Y27632 | STEMCELL Technologies | 72304 |
| PI3k inhibitor LY294002 | Selleckchem | S1105 |
| STEMdiff™ Cardiomyocyte Differentiation Kit | STEMCELL Technologies | 05010 |
| STEMdiff™ Cardiomyocyte Maintenance Kit | STEMCELL Technologies | 05020 |
| STEMdiff™ Cardiomyocyte Dissociation Kit | STEMCELL Technologies | 05025 |
| CryoStor® CS10 | STEMCELL Technologies | 07930 |
| Matrigel® hESC-Qualified Matrix, LDEV-free | Corning | 354277 |
| 5-oxo-ETE | Tocris Bioscience | 1796 |
| Calcitriol | Tocris Bioscience | 2551 |
| Chenodeoxycholic Acid-3-β-D-glucuronide | Toronto Research Chemicals | C291920 |
| In Situ Cell Death Detection Kit, Fluorescein | Roche | 11684795910 |
| truChIP® Chromatin Shearing Kit | Covaris | PN 520237, 520238 |
| Magna ChIP™ A - Chromatin Immunoprecipitation Kit | Sigma-Aldrich | 17-610 |
|  |  |  |
| Trans-Blot® Turbo™ Transfer Starter System, Midi PVDF | Bio-rad | 17001919 |
| miRNeasy mini kit | Qiagen | 217004 |
| **Experimental Models: Cell Lines** | | |
| Human iPSC lines from Down Syndrome (N and P hiPSC cell clones) | This paper | N/A |
| Human ESC H9 cell line | Donated by Dr. Xiaohong Li in Guangdong Provincial People’s Hospital | N/A |
|  |  |  |
| **Software** | | |
| Image J | National Institutes of Health | https://imagej.nih.gov/ij/ |
| Flow Jo | Becton, Dickinson and Company | https://www.flowjo.com/ |
| ZEISS ZEN microscope software for light microscopy systems | ZEISS | https://www.zeiss.com/microscopy/us/products/microscope-software/zen-lite.html |
| THE GENE ONTOLOGY RESOURCE | N/A | http://geneontology.org/ |
| Reactome Pathway Database | N/A | https://reactome.org/ |
| GraphPad Prism | GraphPad | https://www.graphpad.com/ |
| Ingenuity Pathway Analysis (IPA) | QIAGEN | https://digitalinsights.qiagen.com/products-overview/discovery-insights-portfolio/analysis-and-visualization/qiagen-ipa/ |

**Supplementary table files:**

Supplementary table 1. Oligonucleotides

Supplementary table 2. genes readcount_RNA-seq to Figure 1

Supplementary table 3. ATAC-seq_peak to Figure 2

Supplementary table 4. Untargeted metabolomics to Figure 3 and Figure 4

Supplementary table 5. Untargeted metabolomics to Figure 5 and Figure 6

Supplementary table 6. genes readcount_RNA-seq to Figure 5 and Figure 6

Supplementary table 7. genes readcount_RNA-seq to Figure 7
